# Research on the changes in the disease burden of nasopharyngeal carcinoma caused by global occupational formaldehyde exposure from 1990 to 2021 and prediction of future trends

**DOI:** 10.3389/fpubh.2025.1624622

**Published:** 2025-08-20

**Authors:** Tingting Duan, Mengyao Qin, Yue Du, Derong Zhu, Xuejun Zhou, Shun Ding, Desheng Wang

**Affiliations:** ^1^Department of Otolaryngology, Fujian Medical University Union Hospital, Fuzhou, China; ^2^Department of Otolaryngology, Head and Neck Surgery, The First Affiliated Hospital, Hainan Medical University, Haikou, China; ^3^ENT Institute and Otorhinolaryngology Department of Eye & ENT Hospital, State Key Laboratory of Medical Neurobiology and MOE Frontiers Center for Brain Science, Fudan University, Shanghai, China

**Keywords:** occupational formaldehyde exposure, nasopharyngeal carcinoma, disease burden, socio-demographic index, disability-adjusted life years

## Abstract

**Background:**

Explore the changes in the burden of nasopharyngeal carcinoma (NPC) caused by occupational formaldehyde exposure from 1990 to 2021, and predict its development trend up to 2050, to provide references for formulating relevant policies and measures.

**Methods:**

Using the Global Burden of Disease database 2021 (GBD 2021), we counted and analyzed the numbers and standardized rates of NPC deaths and disability–adjusted life years (DALYs) caused by occupational formaldehyde exposure globally, in 21 regions, and in 204 countries. Combined with correlation analysis, health inequality analysis, and frontier analysis, we further comprehensively described the disease burden and its changing trends. In addition, we used the Bayesian age–period–cohort model to predict the disease burden from 2022 to 2050.

**Results:**

From 1990 to 2021 and from 2022 to 2050, the global disease burden of NPC caused by occupational formaldehyde exposure shows a downward trend. In terms of gender, the disease burden is higher among men than among women. From the perspective of the socio-demographic index (SDI), the disease burden is most severe in regions with lower SDI. Geographically, there are significant differences in the disease burden among different countries and regions, with South Asia, East Asia, and Africa having the highest disease burden. In addition, in terms of age distribution, the disease burden is relatively higher among middle-aged people, especially those aged 45–49.

**Conclusions:**

From 1990 to 2021, the disease burden of NPC caused by global occupational formaldehyde exposure showed a downward trend, and the regional burden decreased with the increase of SDI.

## 1 Introduction

Nasopharyngeal carcinoma (NPC), which is a highly prevalent malignant tumor, shows significant differences in terms of incidence and mortality rates worldwide. This phenomenon is particularly prominent in Southeast Asian regions (1). The occurrence of NPC is closely related to multiple factors, such as genetics, environment, and lifestyle (2). Research indicates that occupational exposure to carcinogens is one of the crucial factors influencing the occurrence of NPC. Particularly among workers who are exposed to high concentrations of formaldehyde, the incidence of NPC rises significantly (3).

In recent years, there has been an increasing focus on the correlation between occupational formaldehyde exposure and various types of cancers. The International Agency for Research on Cancer classifies formaldehyde as a carcinogen. It is mainly linked to NPC and leukemia (3). In high-incidence regions, relevant studies on occupational exposure have indicated the potential role of formaldehyde in the pathogenesis of NPC. Nevertheless, current studies still possess certain limitations, especially in terms of the comprehensiveness and representativeness of epidemiological data (4).

Although existing studies have indicated the potential connection between formaldehyde and NPC, systematic evaluations in this domain still lack adequacy. Certain studies have emphasized that occupational exposure may impact the occurrence of NPC through multiple pathways. For instance, it can induce oxidative stress responses and cause genomic instability in cells (5). Consequently, conducting a systematic evaluation of the relationship between occupational formaldehyde exposure and NPC is beneficial in filling the research void in this field and offering a scientific foundation for the formulation of public health policies.

This research utilizes the method of evaluation and comparison by leveraging Global Burden of Disease (GBD) data. It analyzes the epidemiological data regarding occupational exposure to formaldehyde and NPC on a global scale. Through the integration of the findings from multiple studies, a more comprehensive risk assessment can be offered, and the biases introduced by individual studies can be mitigated. This approach not only furnishes crucial information for the formulation of public health policies but also stimulates further research in relevant fields, leads to the improvement of occupational safety standards, and safeguards the health of high-risk populations (6). By systematically evaluating occupational exposure to formaldehyde, the research will lay a scientific foundation for the prevention and control strategies of NPC and enhance the public's understanding and attention to this disease.

## 2 Methods

### 2.1 Data sources

The research data is sourced from GBD 2021 (https://vizhub.healthdata.org). It encompasses mortality and morbidity rates for 204 countries and regions (including subnational estimates in 21 countries and regions), 288 causes of death, 371 diseases and injuries, and 88 risk factors (7). In this study, relevant data on NPC deaths and disability–adjusted life years (DALYs) resulting from occupational exposure to formaldehyde risk factors from 1990 to 2021 were selected for analysis. DALYs serve as a measure of disease burden and are expressed in terms of the number of years of life lost due to poor health, disability, or premature death. Socio-Demographic Index (SDI) is a comprehensive indicator reflecting the development status of a country or region. GDB 2021 classifies 204 countries and 21 regions into five categories, namely low, low-middle, middle, middle-high, and high SDI (8).

### 2.2 Statistical analysis

All statistical evaluations and data representations were conducted utilizing R (version 4.4.2) and JD_GBDR (V2.45.1, Jingding Medical Technology Co., Ltd.).

Describe the burden of NPC attributed to occupational exposure to formaldehyde in different years, among different genders, and for various age groups. In order to eliminate the impact of differences in age distribution on the analysis results, this study adopts the direct method. It standardizes the age based on the world standard population age structure as reported in the GBD 2021 report. The calculation formula is as follows: the age-standardized rate (ASR) = ∑ (the incidence rate of each age group × the proportion of the corresponding age group in the standard population) (9).

We utilize the linear regression model to fit the years and the ASR after performing a logarithmic transformation. Subsequently, we calculate the estimated annual percentage changes (EAPC) by using the slope of the model. If the 95% confidence interval (CI) of EAPC is >0, it can be concluded that the ASR exhibits an upward trend. Conversely, when the 95% CI of EAPC is < 0, it indicates that the ASR shows a downward trend. In addition, if the 95% CI is equal to 0, it means that the ASR is in a stable state (10).

In order to conduct a profound analysis of the association mechanism between the level of social development and the disease burden, this study establishes a two-dimensional analysis framework. Firstly, Spearman correlation analysis is employed to expose the strength of the association between the SDI and each standardized rate value, and the correlation is quantified by the *R* value.

After having clarified the present trend and influencing factors, this research makes use of preface analysis methods to evaluate the optimization space of disease burden. By referring to the optimal practice boundary constructed by SDI, the “effective difference” between the observed values and the theoretical optimal values of each country or region is determined. Here, the “effective difference” particularly refers to the distance between the points represented by each country or region and the boundary. It shows the gap between the observed disease burden and the potentially achievable lowest disease burden in a country or region under its specific SDI circumstances. This indicator is capable of reflecting the potential for reducing disease burden that can be achieved through optimizing resource allocation under specific social and demographic conditions (11).

In order to assess the health equity of the system, this study incorporates two types of complementary indicators. Firstly, the inequality slope index directly measures the absolute burden difference between the extreme groups of SDI. A positive value implies that the burden is concentrated in high SDI countries, while a negative value indicates that the burden is prominent in low SDI countries. Moreover, the absolute value is positively correlated with the degree of inequality. Secondly, the concentration index is calculated by integrating the Lorenz concentration curve (with the interval ranging from −1 to 1). A negative value suggests that the health burden is concentrated in low SDI countries, and the absolute value reflects the degree of unfairness in social stratification (12).

In order to predict disease indicators from 2022 to 2050, a Bayesian age-period-cohort model is constructed. This model integrates prior information and sample data to obtain the posterior distribution of parameters. Subsequently, it presents estimation results, which include a 95% uncertainty interval (UI). This specific model is extremely suitable for dealing with data that has time-dependent and population structure variations. It can successfully control estimation errors. We consider *P* < 0.05 as a statistically significant difference (13).

## 3 Results

### 3.1 Global burden of disease

From a global standpoint, during the period from 1990 to 2021, by referring to ASR data, it was observed that the DALYs resulting from NPC caused by occupational exposure to formaldehyde decreased from 0.45 years per 100,000 people to 0.30 years. This demonstrated a remarkable downward trend, with an EAPC of −1.83%. It is important to note that although the deaths remained relatively stable within this period, at 0.01 cases per 100,000 people, the EAPC was −1.73%, suggesting a slight improvement trend (Table 1).

**Table 1:** ASR deaths, DALYs and EAPC of NPC caused by occupational formaldehyde exposure in the global, SDI and regional level from 1990 to 2021.

**location_name**	**Deaths**			**DALYs**		
	**1990 (per 100,000 population, 95 % UI)**	**2021** (**per 100,000 population, 95 % UI)**	**EAPCs (95 % CI)**	**1990 (per 100,000 population, 95 % UI)**	**2021 (per 100,000 population, 95 % UI)**	**EAPCs (95 % CI)**
Global	0.01 (0.01, 0.01)	0.01 (0.00, 0.01)	−1.73 (−1.94, −1.53)	0.45 (0.30, 0.62)	0.30 (0.19, 0.44)	−1.83 (−2.04, −1.62)
High SDI	0.00 (0.00, 0.00)	0.00 (0.00, 0.00)	−1.81 (−1.94, −1.68)	0.12 (0.08, 0.17)	0.08 (0.05, 0.11)	−1.93 (−2.07, −1.79)
High-middle SDI	0.01 (0.01, 0.02)	0.01 (0.01, 0.02)	−2.06 (−2.35, −1.76)	0.66 (0.42, 0.94)	0.45 (0.29, 0.69)	−2.11 (−2.43, −1.78)
Middle SDI	0.02 (0.01, 0.02)	0.01 (0.01, 0.01)	−2.30 (−2.53, −2.08)	0.70 (0.46, 0.97)	0.38 (0.25, 0.54)	−2.41 (−2.64, −2.19)
Low-middle SDI	0.01 (0.00, 0.01)	0.01 (0.00, 0.01)	0.01 (−0.05, 0.06)	0.25 (0.16, 0.36)	0.25 (0.16, 0.36)	−0.05 (−0.10, 0.00)
Low SDI	0.00 (0.00, 0.01)	0.00 (0.00, 0.01)	−0.51 (−0.65, −0.37)	0.22 (0.14, 0.31)	0.20 (0.12, 0.31)	−0.55 (−0.69, −0.42)
Andean Latin America	0.00 (0.00, 0.00)	0.00 (0.00, 0.00)	−0.13 (−0.38, 0.12)	0.04 (0.03, 0.05)	0.03 (0.02, 0.05)	−0.29 (−0.54, −0.03)
Australasia	0.00 (0.00, 0.00)	0.00 (0.00, 0.00)	−2.20 (−2.28, −2.11)	0.03 (0.02, 0.05)	0.02 (0.01, 0.03)	−2.13 (−2.23, −2.04)
Caribbean	0.00 (0.00, 0.00)	0.00 (0.00, 0.00)	1.62 (1.51, 1.72)	0.08 (0.05, 0.11)	0.12 (0.08, 0.17)	1.56 (1.45, 1.67)
Central Asia	0.00 (0.00, 0.00)	0.00 (0.00, 0.00)	1.03 (0.76, 1.30)	0.08 (0.05, 0.11)	0.11 (0.07, 0.15)	1.05 (0.78, 1.32)
Central Europe	0.00 (0.00, 0.00)	0.00 (0.00, 0.00)	−0.27 (−0.56, 0.02)	0.03 (0.02, 0.04)	0.03 (0.02, 0.04)	−0.47 (−0.75, −0.18)
Central Latin America	0.00 (0.00, 0.00)	0.00 (0.00, 0.00)	−0.78 (−0.91, −0.65)	0.06 (0.04, 0.08)	0.05 (0.03, 0.07)	−0.79 (−0.90, −0.68)
Central Sub-Saharan Africa	0.00 (0.00, 0.00)	0.00 (0.00, 0.00)	−0.58 (−0.77, −0.40)	0.07 (0.04, 0.11)	0.06 (0.04, 0.10)	−0.57 (−0.75, −0.38)
East Asia	0.03 (0.02, 0.04)	0.02 (0.01, 0.02)	−2.75 (−3.06, −2.45)	1.29 (0.86, 1.80)	0.69 (0.43, 1.05)	−2.77 (−3.09, −2.44)
Eastern Europe	0.00 (0.00, 0.00)	0.00 (0.00, 0.00)	−0.82 (−1.11, −0.53)	0.02 (0.01, 0.03)	0.02 (0.01, 0.03)	−0.81 (−1.11, −0.51)
Eastern Sub-Saharan Africa	0.01 (0.00, 0.01)	0.01 (0.01, 0.01)	0.27 (0.13, 0.41)	0.33 (0.21, 0.47)	0.38 (0.23, 0.59)	0.21 (0.07, 0.36)
High-income Asia Pacific	0.00 (0.00, 0.00)	0.00 (0.00, 0.00)	−1.52 (−1.82, −1.22)	0.03 (0.02, 0.05)	0.02 (0.01, 0.03)	−1.78 (−2.07, −1.49)
High-income North America	0.00 (0.00, 0.00)	0.00 (0.00, 0.00)	−2.08 (−2.20, −1.96)	0.03 (0.02, 0.04)	0.02 (0.01, 0.02)	−2.04 (−2.16, −1.92)
North Africa and Middle East	0.00 (0.00, 0.01)	0.00 (0.00, 0.00)	−1.24 (−1.28, −1.19)	0.18 (0.11, 0.26)	0.12 (0.08, 0.18)	−1.31 (−1.35, −1.26)
Oceania	0.00 (0.00, 0.00)	0.00 (0.00, 0.00)	0.16 (0.08, 0.24)	0.12 (0.07, 0.19)	0.12 (0.07, 0.20)	0.16 (0.08, 0.23)
South Asia	0.01 (0.00, 0.01)	0.01 (0.00, 0.01)	−0.87 (−0.98, −0.76)	0.32 (0.19, 0.46)	0.25 (0.16, 0.37)	−0.92 (−1.01, −0.82)
Southeast Asia	0.01 (0.01, 0.02)	0.01 (0.01, 0.02)	0.08 (0.00, 0.15)	0.53 (0.35, 0.73)	0.55 (0.36, 0.78)	−0.03 (−0.12, 0.06)
Southern Latin America	0.00 (0.00, 0.00)	0.00 (0.00, 0.00)	−2.07 (−2.31, −1.83)	0.10 (0.07, 0.15)	0.05 (0.03, 0.07)	−2.05 (−2.30, −1.79)
Southern Sub-Saharan Africa	0.00 (0.00, 0.00)	0.00 (0.00, 0.00)	−2.00 (−2.27, −1.73)	0.12 (0.08, 0.16)	0.07 (0.05, 0.10)	−1.83 (−2.11, −1.54)
Tropical Latin America	0.00 (0.00, 0.00)	0.00 (0.00, 0.00)	−0.33 (−0.88, 0.22)	0.07 (0.05, 0.10)	0.07 (0.05, 0.10)	−0.36 (−0.91, 0.19)
Western Europe	0.00 (0.00, 0.00)	0.00 (0.00, 0.00)	−2.96 (−3.05, −2.88)	0.04 (0.03, 0.06)	0.02 (0.01, 0.02)	−3.08 (−3.17, −2.98)
Western Sub-Saharan Africa	0.00 (0.00, 0.00)	0.00 (0.00, 0.00)	−0.35 (−0.57, −0.14)	0.12 (0.07, 0.17)	0.11 (0.06, 0.18)	−0.30 (−0.51, −0.09)

### 3.2 Regional burden of disease

After the stratification based on SDI, distinct trends can be noticed. In the high SDI area, the DALYs witnessed a decrease from 0.12 to 0.08, accompanied by an EAPC of −1.93%, demonstrating the most remarkable improvement. In contrast, in the low SDI region, the DALYs slightly decreased from 0.22 to 0.20, with an EAPC of −0.55%, indicating that although there was an improvement, the pace was relatively slower. Moreover, the mortality rate in the high SDI region has been kept at a very low level without notable variations. Similarly, the deaths in the low SDI region also exhibited small changes (Table 1 and Supplementary Figure S1).

Specifically, when it comes to 21 regions, their change patterns become more complex. A larger number of regions exhibit a downward trend. Take East Asia as an example. In this region, significant achievements have been attained in reducing the DALYs of NPC caused by occupational formaldehyde exposure. The DALY value has dropped significantly from 1.29 to 0.69 years, and the Estimated EAPC reached −2.77%. Meanwhile, the deaths also showed a notable downward trend. The Western European region has achieved extremely remarkable results in reducing both DALYs and deaths. The EAPCs are −3.08% and −2.96%, respectively. However, some regions show an upward trend. In the Caribbean region, the DALYs actually increased from 0.08 to 0.12, and the EAPC was as high as 1.56%; the deaths EAPC was 1.62%. The Central Asian region, the Eastern Sub-Saharan Africa region, and the Oceania region all demonstrated the same trend (Table 1 and Supplementary Figure S1).

### 3.3 204 countries' burden of disease

**Figure 1:**
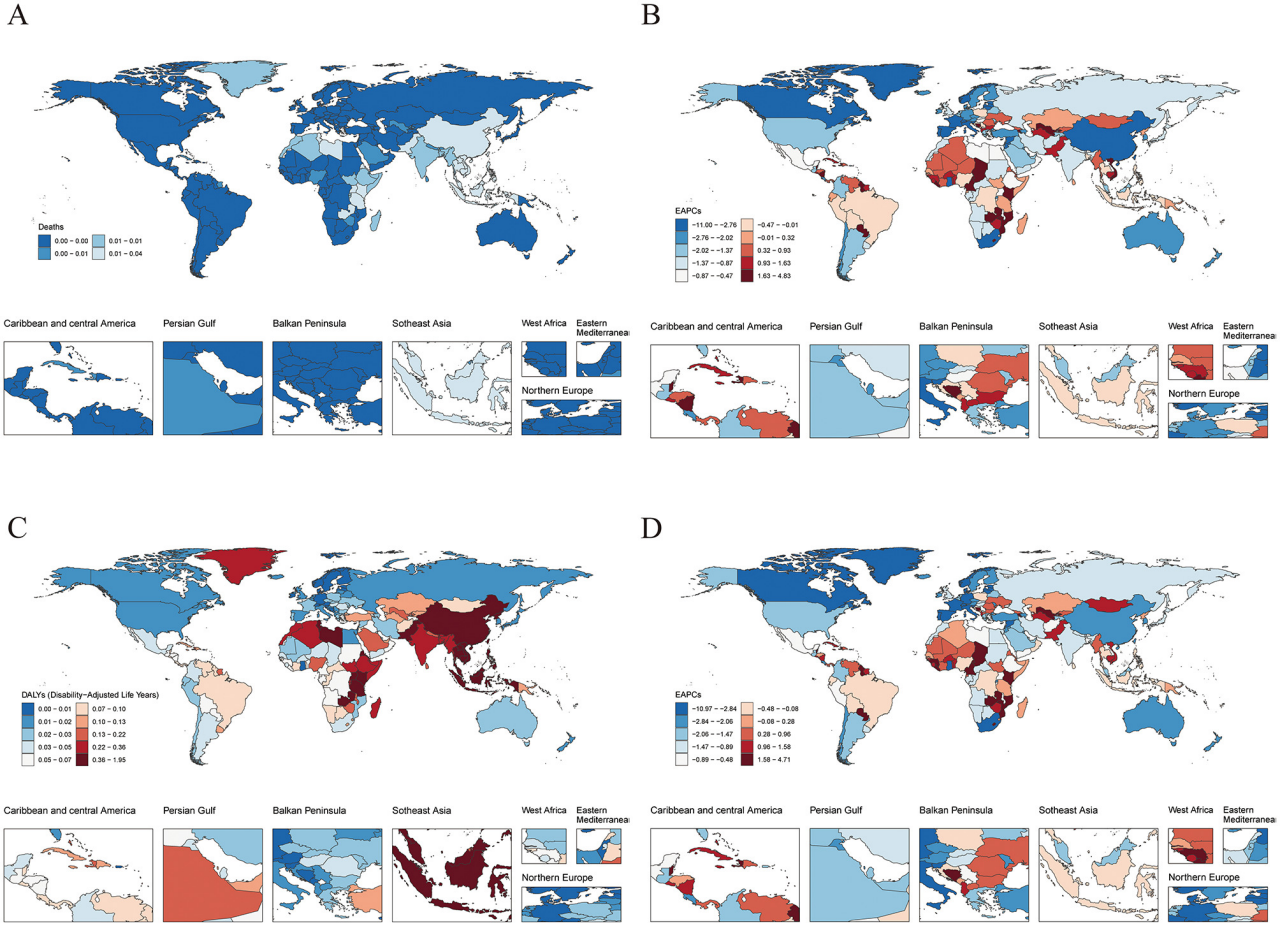
Global burden of disease across 204 countries and territories in 2021. **(A)** Age-standardized deaths. **(B)** EAPC of age-standardized deaths. **(C)** Age-standardized DALYs. **(D)** EAPC of age-standardized DALYs.

Among the total 204 countries worldwide, the health burden associated with NPC resulting from occupational formaldehyde exposure shows significant differences. Regarding deaths, based on the 2021 data, Malaysia has the highest rate, with 0.04 deaths per 100,000 people (95% UI: 0.03–0.07), while several countries, such as Afghanistan and Albania, report zero deaths. In terms of disease burden (DALYs), Malaysia takes the lead with 1.93 years per 100,000 people (95%UI: 1.17–2.86). Likewise, there are several countries that report zero (as shown in Figures 1A, C). Trend analysis indicates that occupational exposure to formaldehyde results in a notable increase in NPC deaths in 79 countries. Among them, the increase is most evident in Cabo Verde. Meanwhile, in one country, there is no significant change. Additionally, 124 countries show a decrease, with the most substantial decrease observed in Ghana (Figure 1B). Regarding DALYs, there is a significant increase in 78 countries, with the largest increase taking place in Cabo Verde. On the other hand, 126 countries experience a decrease, and the most significant decrease is noted in Ghana (Figure 1D). A detailed presentation of the disease burden data for all 204 countries and regions is provided in Supplementary Table S1.

### 3.4 Age-sex-time trends in the burden of disease

**Figure 2:**
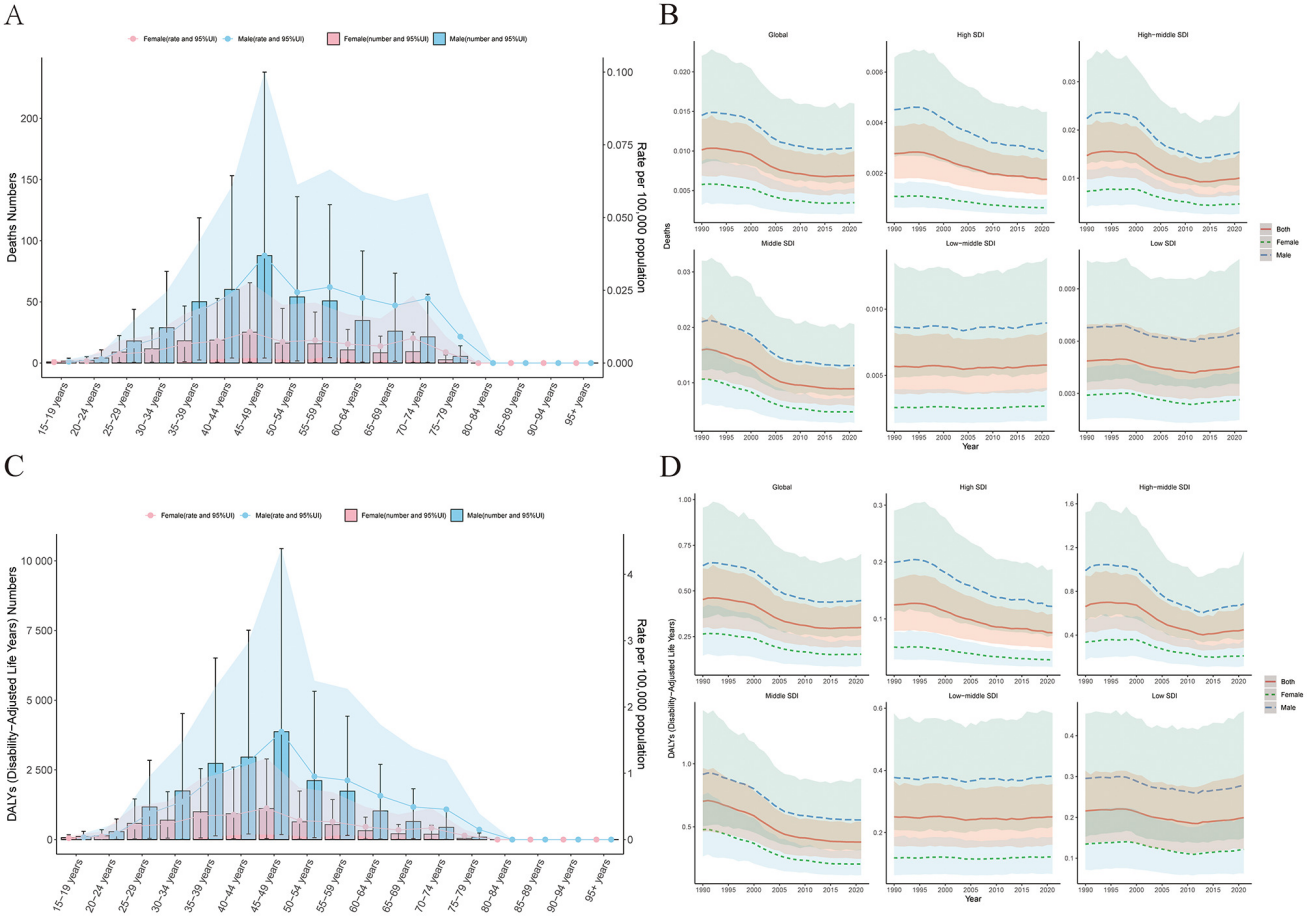
Age-sex-time trends in disease burden. **(A)** Age and sex trends in deaths. **(B)** Sex-time trends in deaths. **(C)** Age and sex trends in DALYs. **(D)** Sex-time trends in DALYs.

In 2021, through age-sex correlation analysis, it was shown that global occupational formaldehyde exposure causes the deaths and DALYs of NPC to increase first and then decrease as age increases. The trends for men and women are similar. It is noteworthy that the disease burden is the greatest in the age group of 45–49. Nevertheless, when looking at the entire age range, the disease burden of men is significantly higher than that of women (Figures 2A, C).

Sex-time correlation analysis indicates that within all gender groups, the global disease burden is experiencing a gradual decline. In low and lower-middle SDI regions, the burden for the entire population (males and females) either fluctuates slightly or shows an increase. In contrast, the other three SDI regions consistently demonstrate a slow downward trend (Figures 2B, D).

### 3.5 Joinpoint regression analysis of occupational formaldehyde exposure and NPC

**Figure 3:**
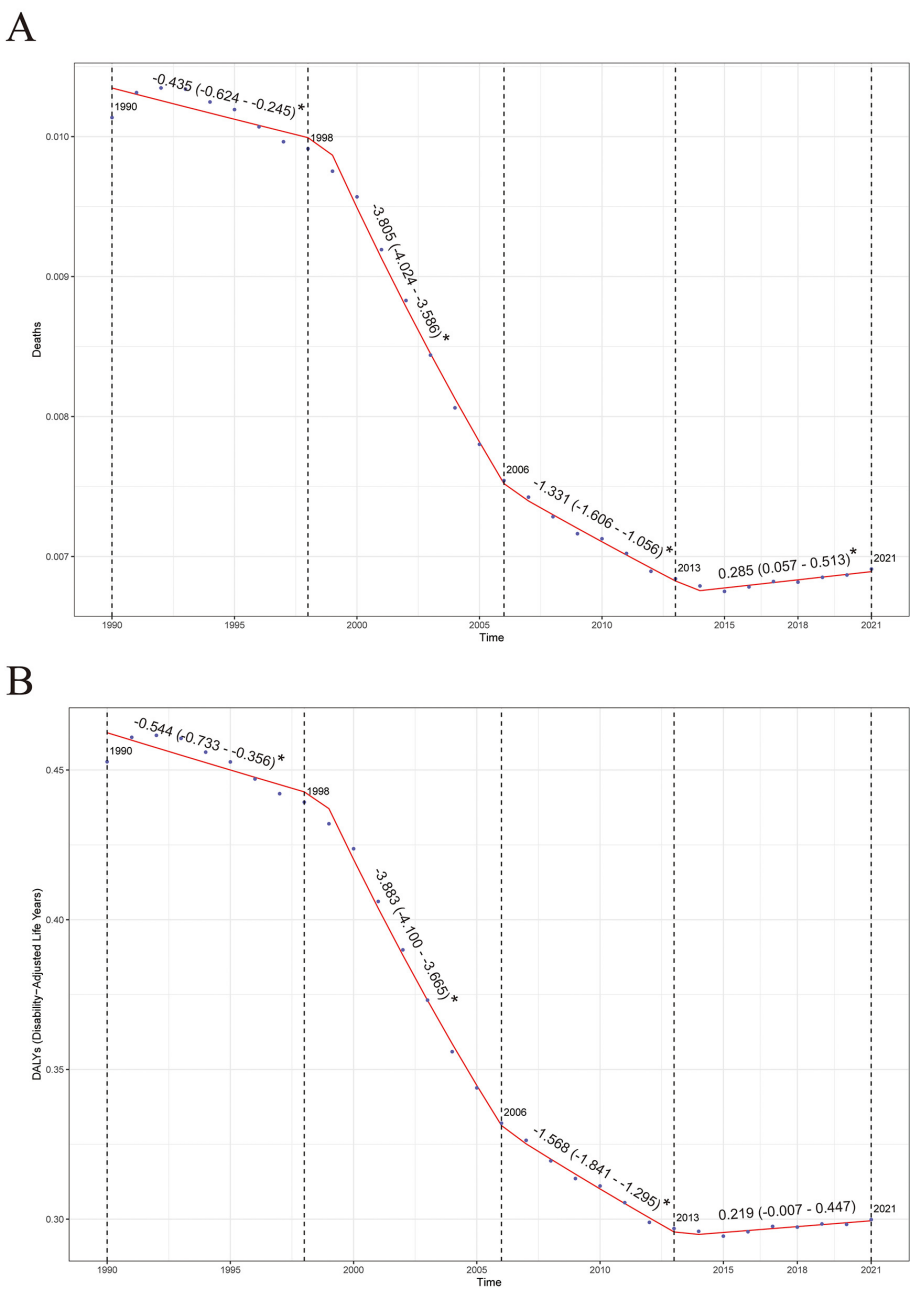
Joinpoint regression analysis results. **(A)** Deaths. **(B)** DALYs. *P < 0.05.

Joinpoint regression analysis shows that during the period from 1990 to 2021, the deaths and DALYs of NPC caused by occupational formaldehyde exposure globally generally showed a downward trend. Specifically, the AAPC of the deaths is −1.29 (95% CI: −1.517, −1.063), and the AAPC of DALYs is −1.38 (95% CI: −1.604, −1.155). In the analysis of deaths and DALYs, three key Joinpoints in 1998, 2006, and 2013 were clearly identified. From 1990 to 1998, and then from 1998 to 2006, as well as from 2006 to 2013, the overall disease burden in these 3-year segments showed a significant downward trend. However, from 2013 to 2021, there was an upward trend (Figure 3).

### 3.6 The relationship between deaths and DALYs and the SDI

**Figure 4:**
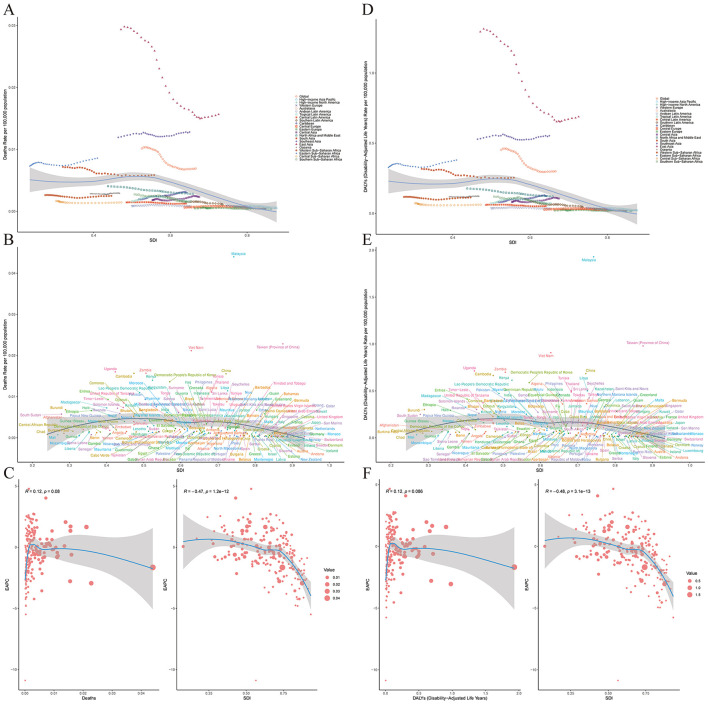
The relationship between the disease burden, EAPC and SDI in 21 regions and 204 countries. **(A)** Deaths and SDI in 21 regions. **(B)** Deaths and SDI in 204 countries. **(C)** EAPC and SDI of deaths in 204 countries. **(D)** DALYs and SDI in 21 regions. **(E)** DALYs and SDI in 204 countries. **(F)** EAPC and SDI of DALYs in 204 countries.

This study explored the associations between NPC deaths and DALYs and different countries' SDI by analyzing global disease burden data. The results showed that in low SDI countries, the deaths and DALYs of NPC were significantly higher than those in high SDI countries (Figures 4A, D), indicating that the improvement of economic development level, public health conditions, and the accessibility of medical services is of great significance for reducing NPC-related health losses. It is worth noting that although the overall trend shows that with the increase of SDI, the burden of NPC has decreased, some middle-income countries such as Malaysia and Vietnam have shown higher deaths and DALYs values of NPC, suggesting that there may be special environmental or genetic risk factors in these regions that need to be further investigated (Figures 4B, E). In addition, statistical analysis found that the correlation coefficient between NPC deaths and SDI was −0.47 (*P* < 0.01), and the correlation coefficient between DALYs and SDI was −0.48 (*P* < 0.01), which confirmed the trend of reducing the burden of NPC with the increase of SDI (Figures 4C, F).

### 3.7 Inequality analysis of disease burden from 1990 to 2021

**Figure 5:**
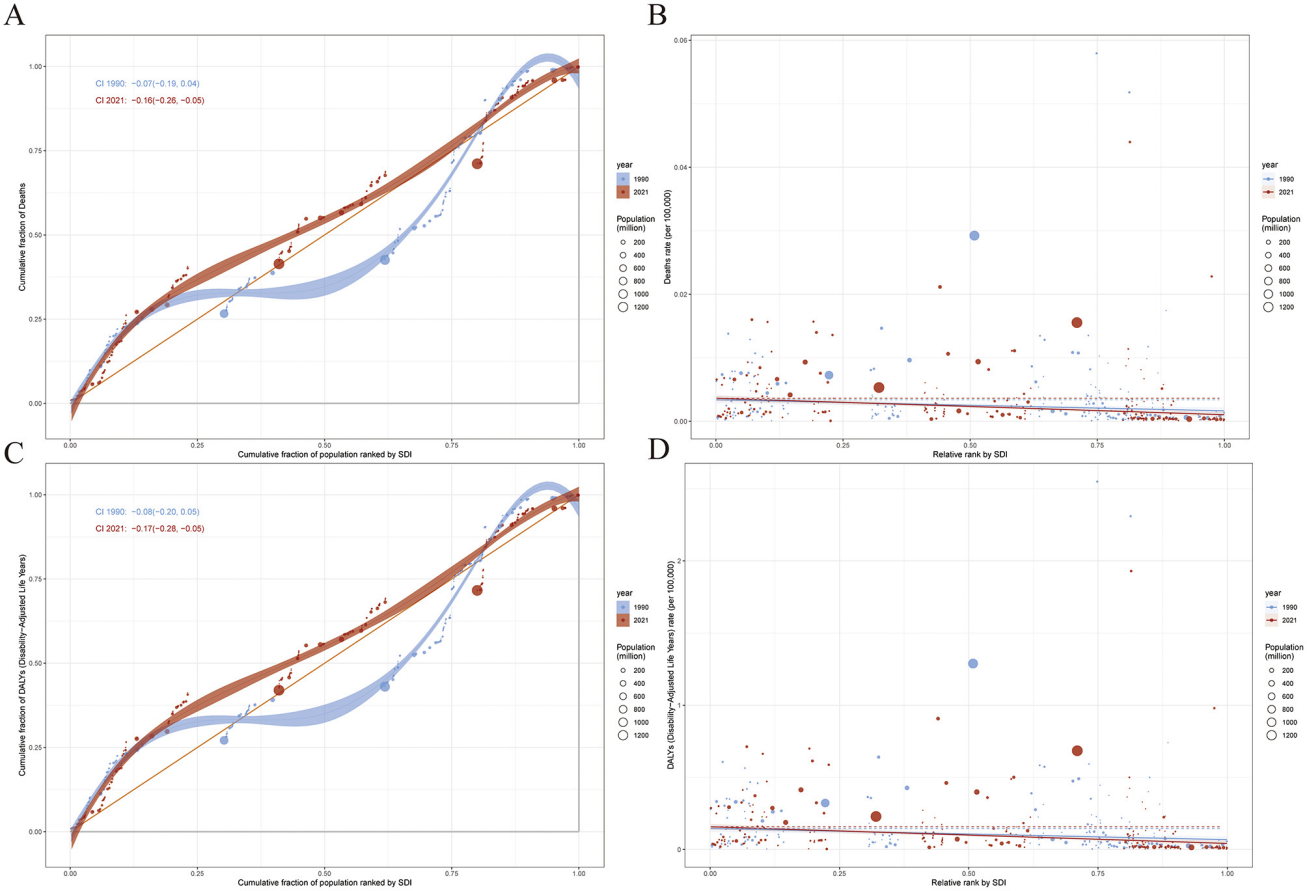
Health inequality regression and concentration curves for the deaths and DALYs of disease in 1990 and 2021. **(A)** The concentration index of deaths. **(B)** The inequality slope index of deaths. **(C)** The concentration index of DALYs. **(D)** The inequality slope index of DALYs.

From 1990 to 2021, occupational formaldehyde exposure among countries has led to health inequality phenomena in NPC's disease burden that are closely related to SDI. Specifically, a higher disease burden is disproportionately concentrated in countries and regions with lower SDI. Over time, the degree of this health inequality has increased. During this period, the concentration indices of deaths and DALYs rates have both risen, increasing from −0.07 and −0.08 in 1990 to −0.16 and −0.17 in 2021 (Figures 5A, C). deaths and DALYs are higher in low SDI areas and lower in high SDI areas, and this trend exists in both 1990 and 2021. However, the deaths and DALYs in 2021 are generally lower than those in 1990, indicating that overall, they have decreased over time (Figures 5B, D).

### 3.8 Frontier analysis of NPC caused by occupational formaldehyde exposure from 1990 to 2021

**Figure 6:**
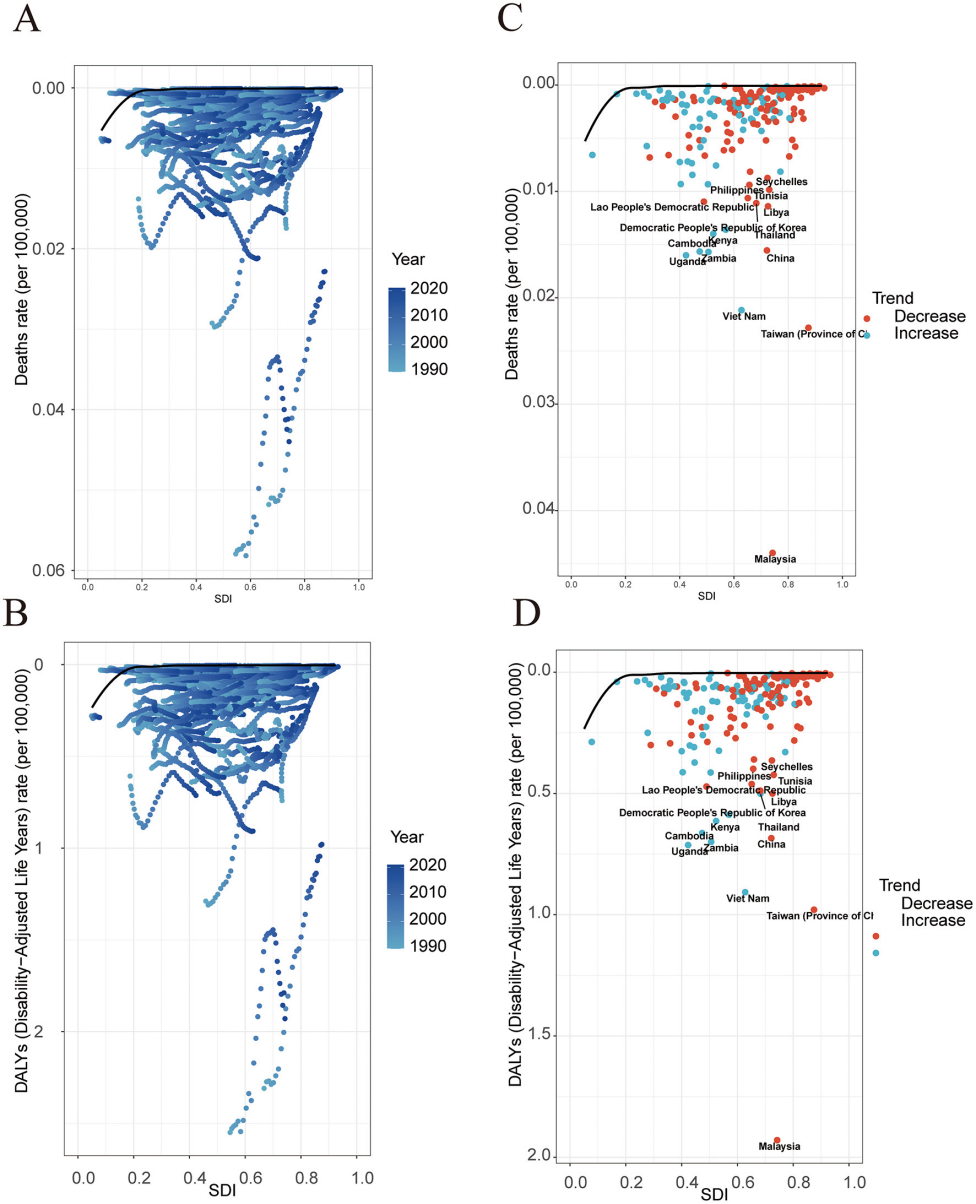
Frontier analysis based on the SDI and ASR of disease burden in 204 countries and regions, 1990–2021. **(A)** Standardized deaths. **(B)** Standardized DALYs. **(C)** Standardized deaths. **(D)** Standardized DALYs.The points between C and D represent countries and regions, and the boundaries are demarcated in pure black. Black fonts are used to label the top 15 countries with the largest significant differences. Red dots indicate that the standardized disease burden rate decreased from 1990 to 2021, while blue dots indicate that the standardized disease burden rate increased from 1990 to 2021.

The results of the frontier analysis (1990–2021) show that the countries and regions with the largest actual gaps (deaths and DALYs) from the theoretical boundary include Malaysia, Taiwan (Province of China), Vietnam, Uganda, and Zambia (Figures 6A, B). Among the 15 countries with the largest differences, they are mainly developing countries located in East Asia, South Asia, and Africa. The disease burden in Asian countries shows a downward trend, while that in Africa shows an upward trend (Figures 6C, D). There is a relatively high “effective difference” compared to their development levels, that is, there is a large gap between the observed disease burden and the potentially achievable minimum disease burden. This indicates that there is still significant room for improvement in these countries or regions in reducing the disease burden. Specifically, the “effective difference” refers to the distance from each country or regional representative point to the best practice boundary. Specifically, it is the gap between the observed disease burden and the lowest disease burden that could be achieved under its specific SDI. This indicator can reflect the potential to reduce the disease burden by optimizing resource allocation under specific social and demographic conditions. For example, in this study, we found that even though some countries have similar socioeconomic conditions, there are still significant differences in the disease burden of NPC caused by occupational formaldehyde exposure among them, which further emphasizes the importance of adopting best practices and preventive strategies.

### 3.8 Global projection of disease burden from 2022 to 2050

**Figure 7:**
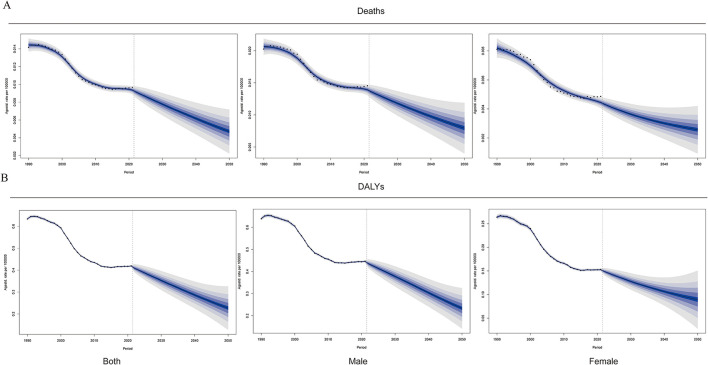
Frontier analysis from 2022 to 2050. **(A)** Rate of both male and female deaths. **(B)** Rate of both, male and female DALYs.

By 2050, the estimated number of NPC deaths and DALYs caused by global occupational formaldehyde exposure is expected to reach 0.013 per 100,000 (95% UI: 0.013–0.014) and 0.162 per 100,000 (95% UI: 0.078–0.246), respectively, and the overall disease burden shows a downward trend. In terms of gender, by 2050, the disease-burden deaths and DALYs of males are expected to reach 0.008 per 100,000 (95% UI: 0.003–0.013) and 0.234 per 100,000 (95% UI: 0.125–0.343), respectively. For females, the disease-burden deaths and DALYs are expected to reach 0.003 per 100,000 (95% UI: 0.001–0.005) and 0.089 per 100,000 (95% UI: 0.016–0.162), respectively. The decline in the disease burden of males is greater than that of females. These predictions indicate that the global disease burden of NPC caused by occupational formaldehyde exposure will continue to decline, but there are still significant gender and regional differences (Figure 7, Supplementary Tables S2, S3).

## 4 Discussion

NPC is a malignancy that arises from the epithelial cells of the nasopharynx, and it is notably associated with various environmental and occupational risk factors, including exposure to formaldehyde (14). This carcinogen is commonly found in industrial settings, leading to significant health concerns for workers in affected industries. The global burden of NPC, particularly due to occupational formaldehyde exposure, has been a subject of increasing scrutiny, as it poses a substantial public health challenge (15). Understanding the epidemiology of NPC, including its incidence, mortality, and DALYs, is crucial for developing effective prevention and intervention strategies aimed at reducing the disease burden associated with this malignancy.

This paper further explores the disease burden of NPC caused by occupational formaldehyde exposure through frontier analysis, health inequality analysis, and predictive analysis. From 1990 to 2021, the age–age-standardized death and DALYs rates of NPC caused by global occupational formaldehyde exposure declined, which is related to the advancement and widespread use of medical diagnostic technology. Moreover, predictive analysis shows that these rates will continue to decline in the next 30 years. In terms of gender, the disease burden is heavier among the male population. Additionally, the analysis results at the SDI, regional, and national levels show that the regions with the heaviest disease burden of NPC caused by occupational formaldehyde exposure are low-SDI regions, East Asia, South Asia, and Africa, respectively. Notably, the overall situation in Africa shows an upward trend. The health inequality analysis reveals that the absolute health inequality of NPC caused by occupational formaldehyde exposure has decreased over the past 32 years, while the relative health inequality has increased. Furthermore, the frontier analysis indicates that even if some countries have similar socioeconomic conditions, there are still significant differences in the disease burden of NPC caused by occupational formaldehyde exposure. This suggests that there is great potential to improve health outcomes by adopting best practices and prevention strategies.

The decline disease burden of NPC caused by occupational formaldehyde exposure is influenced by multiple factors. On the one hand, internationally, many countries and regions have established occupational safety standards for formaldehyde. For example, the Occupational Safety and Health Administration (OSHA) in the United States has set exposure limits for formaldehyde and requires the implementation of strict monitoring and control measures in the workplace to protect the health of workers (16). Secondly, personal protective equipment (PPE) plays a crucial role in reducing formaldehyde exposure. Research shows that the proper use of PPE can significantly reduce workers' formaldehyde exposure in high-risk environments. For example, in a study of the beauty industry, wearing appropriate protective gloves and masks can effectively reduce workers' exposure to formaldehyde and other volatile organic compounds (VOCs) (17). On the other hand, health monitoring and early screening are important strategies for preventing diseases related to formaldehyde exposure. Through regular health examinations, health problems caused by formaldehyde exposure can be detected early, so that corresponding intervention measures can be taken (18). For example, for workers who work in high-risk environments, a systematic health monitoring program should be established, including pulmonary function tests and blood tests, to assess their health status (19). Overall, the hazards of occupational formaldehyde exposure cannot be ignored. Multi-party collaboration is needed to promote relevant research and intervention measures, and ultimately achieve a healthy working environment and a healthy population. At the same time, men bear a heavier disease burden mainly because formaldehyde is a widely used chemical substance, commonly found in the construction, manufacturing, and medical industries. Due to their career choices and work environments, men often face a higher risk of formaldehyde exposure (20). In addition, when choosing careers, men may be more inclined to high-intensity and high-risk work environments, which expose them to higher health risks when being exposed to harmful substances such as formaldehyde (21). Finally, due to long-standing systematic inequalities, different structural barriers, and disparities in the social and economic determinants of health among different countries and regions around the world, the correlation between the SDI and the disease burden has emerged, and health inequalities have widened. However, there is still substantial room for improvement (22).

The impact of structural determinants on the inequality of disease burden. Differences in access to healthcare are important structural determinants that affect health outcomes. Many low-income families have difficulty accessing necessary medical services due to financial burdens, lack of health insurance, or inconvenient transportation. This situation not only leads to delayed diagnosis and treatment of diseases but also exacerbates health inequalities, especially in diseases related to occupational exposure such as NPC (6). In addition, structural deficiencies in the healthcare system, such as uneven distribution of medical services and discrimination against low-income groups, further exacerbate this problem (18). Insufficient enforcement of occupational safety is an important factor leading to an increase in health risks in the workplace. Although many countries have relevant laws and regulations to protect the health and safety of workers, there are often loopholes and deficiencies in actual implementation (16). Especially in low-income and high-risk work environments such as construction and manufacturing, workers often face inadequate safety training and a lack of necessary protective equipment, making them more vulnerable to harmful substances such as carcinogens like formaldehyde (23). In addition, the relationship between socioeconomic factors and health outcomes is complex and profound. Research shows that countries with low SDI are closely related to poorer health outcomes, including higher disease incidence and mortality (18). For example, in the study of NPC, groups with poorer economic conditions often lack health education and prevention knowledge, resulting in low participation in early screening and treatment, thus increasing the burden of the disease (6). Therefore, improving socioeconomic conditions and providing more equitable medical resources are important strategies for reducing health inequalities.

The inflection points observed in the Joinpoint regression analysis may reflect the combined effects of several key factors. First, from a policy perspective, these years coincide with major updates to occupational safety regulations in many countries. For instance, in the U.S., OSHA updated formaldehyde exposure limits during this period and mandated stricter monitoring and control measures in workplaces to protect workers' health (16). Second, industrial restructuring may also have played a role. With the acceleration of globalization, high-formaldehyde-risk industries may have undergone geographic relocation or technological upgrades. For example, labor-intensive manufacturing sectors shifted from developed to developing countries, potentially altering regional patterns of formaldehyde exposure (24). Lastly, advancements in health surveillance and early screening during these years may have improved the detection and management of formaldehyde-related health issues. Regular health check-ups, including lung function tests and blood biomarker analyses, could have facilitated earlier diagnosis and intervention among high-risk workers (25).

Currently, the results show that the disease burden of NPC caused by occupational formaldehyde exposure mainly falls on the middle-aged population, especially those aged 45–49 (3). Decision-makers should fully consider factors such as gender, age, SDI level, and region, and formulate more targeted individualized medical care. Access to and acceptance of nursing care are important predictive factors for preventing NPC caused by formaldehyde (26). Medical resources should be allocated more reasonably, the capacity of health care should be improved, and the medical infrastructure and professional personnel in regions with lower SDI should be increased (27). At the same time, emphasize the importance of multidisciplinary cooperation to improve regional health conditions and expand the scope of disease monitoring and screening.

In the frontier analysis, in countries and regions with large actual gaps, such as Malaysia, Taiwan (China), Vietnam, Uganda, and Zambia, road traffic safety and environmental health problems are more prominent. On the one hand, there are generally problems such as serious road congestion, complex traffic participants (mixed traffic of various means of transportation), and imperfect infrastructure in these areas, which may exacerbate the incidence of road traffic injuries; on the other hand, with the acceleration of the industrialization and urbanization processes, the exposure levels of environmental risk factors such as air pollution and formaldehyde are also rising, becoming important factors affecting public health. It is worth noting that when assessing such health burdens, efforts should be made to distinguish the contributions between environmental exposure and occupational exposure in order to provide a basis for formulating more targeted intervention measures.

This study presents a comprehensive analysis of the global burden of disease associated with NPC due to occupational exposure to formaldehyde, revealing significant trends and disparities across various regions and socioeconomic contexts. However, several limitations must be acknowledged. We acknowledge that the reliance on aggregate data may obscure localized variations in disease burden and exposure levels, potentially leading to an underestimation or overestimation of the true impact in specific populations. Particularly in low-income countries with limited health infrastructure, the inherent uncertainties in the data may affect the robustness of our findings. For instance, assessments of the disease burden caused by occupational formaldehyde exposure in regions with weaker health monitoring systems may have limitations (28). Therefore, we emphasize that future research should aim to incorporate more granular data and consider a broader range of risk factors to better understand the burden of NPC and inform targeted interventions.

Furthermore, not all potential important risk factors and covariates were included in the analysis. Additionally, the widespread impacts of the COVID-19 pandemic on global health conditions and related risk factors have not been systematically quantified and integrated into this version. Therefore, when interpreting the findings, one should consider the current epidemiological context and cautiously assess their applicability and generalizability.

In this study, we relied on occupational formaldehyde exposure data from the GBD 2021 database for our analysis. These data are primarily estimated based on job classifications rather than actual exposure levels obtained through individual biomonitoring. While this job-based approach facilitates a comprehensive assessment of the global burden of NPC associated with formaldehyde exposure, it does have certain limitations. Individual variations—such as differences in actual exposure concentrations among workers in the same occupation due to varying workplace environments, ventilation conditions, and PPE usage—are not accounted for. As a result, exposure estimates based solely on job classification may lead to either underestimation or overestimation of the true exposure levels. This not only affects the accuracy of understanding the relationship between formaldehyde exposure and NPC but may also introduce bias in estimating disease burden in specific regions or populations.

## 5 Conclusions

The results of this study show that from 1990 to 2021, the global disease burden of NPC caused by occupational formaldehyde exposure has been on a downward trend. In terms of gender, the disease burden is higher among men than women. From the perspective of the SDI, the disease burden is most significant in regions with low SDI. Geographically, there are significant differences in the disease burden among different countries and regions. Additionally, in terms of age distribution, the disease burden is relatively high among middle-aged people, especially those aged 45–49. Based on the above research results, we should pay attention to occupational formaldehyde exposure throughout the entire life cycle, accelerate the formulation of relevant policies and the implementation of protective measures, and strive to reduce the occurrence and progression of the disease, so as to effectively alleviate the disease burden of NPC.

## Data Availability

The original contributions presented in the study are included in the article/Supplementary material, further inquiries can be directed to the corresponding authors.
