# Supplementary material for: Research on the changes in the disease burden of nasopharyngeal carcinoma caused by global occupational formaldehyde exposure from 1990 to 2021 and prediction of future trends
Source: Front Public Health. 2025 Aug 20;13:1624622. doi: 10.3389/fpubh.2025.1624622 (PMC12404924; doi:10.3389/fpubh.2025.1624622)
Supplement: Supplementary file 1 [file Table_1.docx]

**
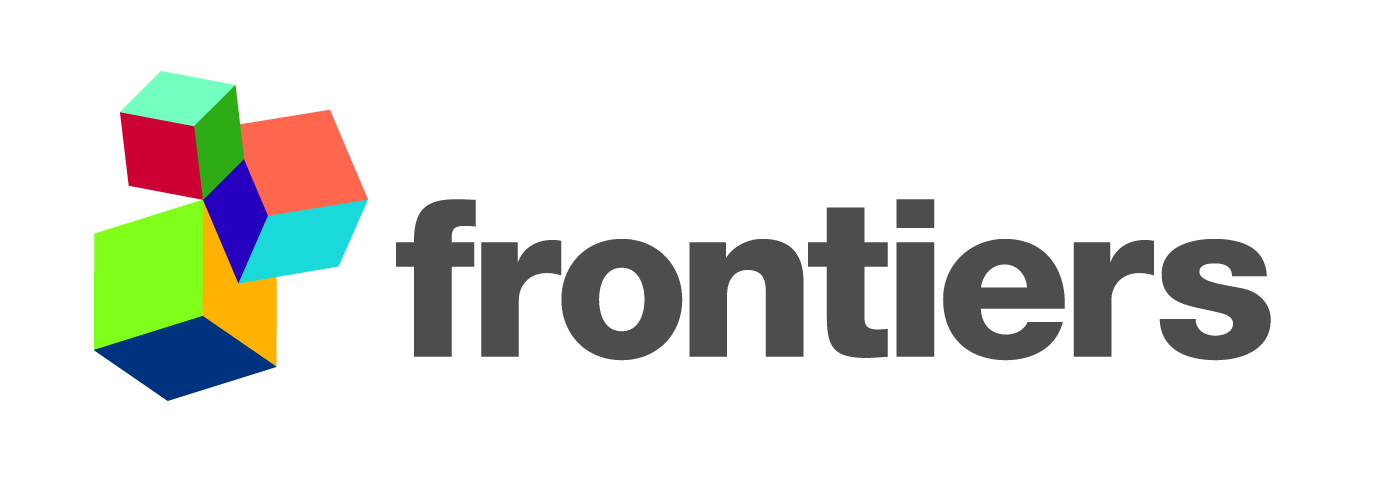
**

Supplementary Material

Research on the Changes in the Disease Burden of Nasopharyngeal Carcinoma Caused by Global Occupational Formaldehyde Exposure from 1990 to 2021 and Prediction of Future Trends

**Table S1:** ASR disease burden across 204 countries and regions.

| **location_name** | **deaths** | | | **DALYs** | | |
| --- | --- | --- | --- | --- | --- | --- |
|  | **1990(per**  **100,000**  **population, 95 %**  **UI）** | **2021(per**  **100,000**  **population, 95 %**  **UI）** | **EAPCs(95 %**  **CI）** | **1990(per**  **100,000**  **population, 95 %**  **UI）** | **2021(per**  **100,000**  **population, 95 %**  **UI）** | **EAPCs(95 %**  **CI）** |
| Afghanistan | 0.00(0.00,0.00) | 0.00(0.00,0.00) | -0.94(-1.12,-0.76) | 0.11(0.04,0.17) | 0.09(0.04,0.14) | -0.81(-0.98,-0.63) |
| Albania | 0.00(0.00,0.00) | 0.00(0.00,0.00) | 1.55(1.19,1.91) | 0.01(0.01,0.02) | 0.02(0.01,0.02) | 1.44(1.04,1.83) |
| Algeria | 0.01(0.01,0.01) | 0.01(0.00,0.01) | 0.35(0.11,0.60) | 0.39(0.22,0.62) | 0.36(0.21,0.55) | 0.25(0.02,0.48) |
| American Samoa | 0.01(0.01,0.01) | 0.01(0.01,0.01) | 0.14(-0.05,0.34) | 0.38(0.22,0.58) | 0.36(0.21,0.55) | 0.20(-0.00,0.41) |
| Andorra | 0.00(0.00,0.00) | 0.00(0.00,0.00) | -1.91(-2.07,-1.75) | 0.02(0.01,0.03) | 0.01(0.00,0.02) | -1.92(-2.08,-1.77) |
| Angola | 0.00(0.00,0.00) | 0.00(0.00,0.00) | -0.97(-1.20,-0.73) | 0.09(0.05,0.14) | 0.07(0.04,0.10) | -0.89(-1.13,-0.65) |
| Antigua and Barbuda | 0.00(0.00,0.00) | 0.00(0.00,0.00) | 0.32(0.19,0.44) | 0.06(0.04,0.09) | 0.06(0.04,0.09) | 0.28(0.14,0.43) |
| Argentina | 0.00(0.00,0.00) | 0.00(0.00,0.00) | -1.95(-2.19,-1.71) | 0.12(0.07,0.17) | 0.05(0.03,0.08) | -1.97(-2.22,-1.72) |
| Armenia | 0.00(0.00,0.00) | 0.00(0.00,0.00) | 2.61(2.03,3.20) | 0.03(0.02,0.04) | 0.05(0.03,0.08) | 2.41(1.80,3.03) |
| Australia | 0.00(0.00,0.00) | 0.00(0.00,0.00) | -2.20(-2.28,-2.11) | 0.04(0.02,0.05) | 0.02(0.01,0.03) | -2.13(-2.22,-2.04) |
| Austria | 0.00(0.00,0.00) | 0.00(0.00,0.00) | -2.15(-2.24,-2.06) | 0.03(0.02,0.04) | 0.01(0.01,0.02) | -2.33(-2.42,-2.23) |
| Azerbaijan | 0.00(0.00,0.00) | 0.00(0.00,0.00) | -2.02(-2.23,-1.82) | 0.06(0.03,0.10) | 0.03(0.02,0.05) | -2.09(-2.33,-1.84) |
| Bahamas | 0.00(0.00,0.00) | 0.00(0.00,0.00) | 1.02(0.76,1.28) | 0.09(0.06,0.12) | 0.13(0.08,0.19) | 1.00(0.74,1.27) |
| Bahrain | 0.00(0.00,0.01) | 0.00(0.00,0.00) | -2.39(-2.66,-2.12) | 0.18(0.11,0.28) | 0.10(0.06,0.17) | -2.30(-2.57,-2.04) |
| Bangladesh | 0.01(0.00,0.01) | 0.01(0.00,0.01) | -0.45(-0.52,-0.38) | 0.33(0.17,0.53) | 0.29(0.14,0.55) | -0.48(-0.56,-0.40) |
| Barbados | 0.00(0.00,0.00) | 0.00(0.00,0.00) | 0.77(0.54,1.01) | 0.10(0.07,0.14) | 0.12(0.08,0.18) | 0.64(0.39,0.90) |
| Belarus | 0.00(0.00,0.00) | 0.00(0.00,0.00) | -1.37(-1.75,-0.99) | 0.03(0.01,0.04) | 0.02(0.01,0.03) | -1.52(-1.90,-1.13) |
| Belgium | 0.00(0.00,0.00) | 0.00(0.00,0.00) | -1.87(-2.09,-1.65) | 0.03(0.02,0.04) | 0.02(0.01,0.03) | -2.05(-2.26,-1.83) |
| Belize | 0.00(0.00,0.00) | 0.00(0.00,0.00) | 2.32(1.75,2.89) | 0.04(0.03,0.05) | 0.08(0.06,0.12) | 2.38(1.82,2.95) |
| Benin | 0.00(0.00,0.00) | 0.00(0.00,0.00) | 1.06(0.90,1.21) | 0.04(0.02,0.06) | 0.06(0.03,0.10) | 1.12(0.96,1.28) |
| Bermuda | 0.00(0.00,0.00) | 0.00(0.00,0.00) | -0.66(-0.78,-0.55) | 0.11(0.07,0.17) | 0.09(0.05,0.13) | -0.67(-0.78,-0.55) |
| Bhutan | 0.01(0.00,0.01) | 0.01(0.00,0.01) | -0.60(-0.77,-0.43) | 0.27(0.15,0.43) | 0.23(0.12,0.44) | -0.67(-0.83,-0.51) |
| Bolivia (Plurinational State of) | 0.00(0.00,0.00) | 0.00(0.00,0.00) | -0.01(-0.35,0.33) | 0.05(0.03,0.07) | 0.06(0.03,0.09) | -0.20(-0.54,0.14) |
| Bosnia and Herzegovina | 0.00(0.00,0.00) | 0.00(0.00,0.00) | 2.24(1.94,2.53) | 0.00(0.00,0.01) | 0.01(0.00,0.01) | 2.01(1.72,2.30) |
| Botswana | 0.00(0.00,0.00) | 0.00(0.00,0.00) | -1.34(-1.73,-0.96) | 0.10(0.05,0.16) | 0.07(0.03,0.12) | -1.12(-1.49,-0.76) |
| Brazil | 0.00(0.00,0.00) | 0.00(0.00,0.00) | -0.37(-0.92,0.18) | 0.07(0.05,0.10) | 0.07(0.05,0.10) | -0.40(-0.95,0.16) |
| Brunei Darussalam | 0.00(0.00,0.01) | 0.00(0.00,0.00) | -0.49(-0.70,-0.28) | 0.19(0.12,0.28) | 0.13(0.09,0.19) | -0.53(-0.74,-0.32) |
| Bulgaria | 0.00(0.00,0.00) | 0.00(0.00,0.00) | 1.15(0.87,1.44) | 0.02(0.01,0.03) | 0.03(0.02,0.04) | 0.96(0.68,1.24) |
| Burkina Faso | 0.00(0.00,0.00) | 0.00(0.00,0.00) | 1.31(1.17,1.45) | 0.03(0.02,0.05) | 0.05(0.03,0.08) | 1.40(1.26,1.54) |
| Burundi | 0.01(0.00,0.01) | 0.01(0.00,0.01) | -0.78(-0.95,-0.61) | 0.35(0.21,0.52) | 0.30(0.16,0.50) | -0.83(-1.00,-0.65) |
| Cabo Verde | 0.00(0.00,0.00) | 0.00(0.00,0.00) | 4.78(2.98,6.61) | 0.01(0.01,0.02) | 0.06(0.03,0.10) | 4.66(2.85,6.50) |
| Cambodia | 0.01(0.01,0.02) | 0.02(0.01,0.02) | 1.34(1.31,1.38) | 0.47(0.29,0.71) | 0.66(0.40,1.02) | 1.24(1.21,1.26) |
| Cameroon | 0.00(0.00,0.00) | 0.00(0.00,0.00) | 2.45(2.27,2.62) | 0.03(0.02,0.05) | 0.07(0.04,0.11) | 2.51(2.33,2.68) |
| Canada | 0.00(0.00,0.00) | 0.00(0.00,0.00) | -2.94(-3.12,-2.76) | 0.03(0.02,0.04) | 0.01(0.01,0.02) | -2.86(-3.06,-2.67) |
| Central African Republic | 0.00(0.00,0.00) | 0.00(0.00,0.00) | -1.04(-1.14,-0.93) | 0.09(0.05,0.15) | 0.07(0.04,0.12) | -1.01(-1.12,-0.90) |
| Chad | 0.00(0.00,0.00) | 0.00(0.00,0.00) | 2.47(2.18,2.75) | 0.02(0.01,0.03) | 0.04(0.02,0.06) | 2.53(2.24,2.81) |
| Chile | 0.00(0.00,0.00) | 0.00(0.00,0.00) | -2.32(-2.66,-1.98) | 0.05(0.03,0.07) | 0.02(0.01,0.03) | -2.33(-2.68,-1.98) |
| China | 0.03(0.02,0.04) | 0.02(0.01,0.02) | -2.78(-3.09,-2.46) | 1.29(0.86,1.80) | 0.68(0.43,1.05) | -2.79(-3.12,-2.46) |
| Colombia | 0.00(0.00,0.00) | 0.00(0.00,0.00) | -1.91(-2.06,-1.76) | 0.08(0.06,0.11) | 0.05(0.03,0.07) | -2.01(-2.14,-1.87) |
| Comoros | 0.00(0.00,0.01) | 0.01(0.00,0.01) | 0.29(-0.02,0.59) | 0.22(0.10,0.37) | 0.26(0.13,0.49) | 0.21(-0.14,0.56) |
| Congo | 0.00(0.00,0.00) | 0.00(0.00,0.00) | -1.34(-1.60,-1.08) | 0.09(0.06,0.14) | 0.07(0.04,0.12) | -1.28(-1.55,-1.02) |
| Cook Islands | 0.00(0.00,0.00) | 0.00(0.00,0.00) | 0.52(0.21,0.82) | 0.04(0.02,0.06) | 0.04(0.02,0.06) | 0.63(0.32,0.94) |
| Costa Rica | 0.00(0.00,0.00) | 0.00(0.00,0.00) | -2.11(-2.48,-1.74) | 0.13(0.08,0.18) | 0.09(0.06,0.13) | -2.19(-2.57,-1.80) |
| Croatia | 0.00(0.00,0.00) | 0.00(0.00,0.00) | -0.14(-0.80,0.52) | 0.02(0.01,0.03) | 0.02(0.01,0.02) | 0.54(0.39,0.69) |
| Cuba | 0.00(0.00,0.00) | 0.00(0.00,0.01) | 1.61(1.48,1.74) | 0.08(0.05,0.12) | 0.13(0.08,0.19) | -0.29(-0.95,0.36) |
| Cyprus | 0.00(0.00,0.00) | 0.00(0.00,0.00) | -2.05(-2.26,-1.84) | 0.01(0.01,0.02) | 0.01(0.00,0.01) | 1.33(1.19,1.47) |
| Czechia | 0.00(0.00,0.00) | 0.00(0.00,0.00) | -2.54(-2.66,-2.41) | 0.04(0.03,0.06) | 0.02(0.01,0.03) | -2.13(-2.37,-1.89) |
| C么te d'Ivoire | 0.00(0.00,0.00) | 0.00(0.00,0.00) | 0.47(0.32,0.62) | 0.07(0.04,0.10) | 0.09(0.04,0.15) | -2.54(-2.66,-2.43) |
| Democratic People's Republic of Korea | 0.01(0.01,0.02) | 0.01(0.01,0.02) | 0.10(0.06,0.15) | 0.57(0.34,0.92) | 0.59(0.36,0.94) | -0.03(-0.09,0.03) |
| Democratic Republic of the Congo | 0.00(0.00,0.00) | 0.00(0.00,0.00) | -0.36(-0.58,-0.14) | 0.06(0.04,0.10) | 0.06(0.03,0.10) | -0.36(-0.58,-0.14) |
| Denmark | 0.00(0.00,0.00) | 0.00(0.00,0.00) | -2.71(-2.91,-2.51) | 0.02(0.01,0.02) | 0.01(0.00,0.01) | -2.84(-3.05,-2.63) |
| Djibouti | 0.00(0.00,0.01) | 0.01(0.00,0.01) | 0.66(0.54,0.78) | 0.18(0.09,0.34) | 0.23(0.10,0.44) | 0.60(0.48,0.72) |
| Dominica | 0.00(0.00,0.00) | 0.00(0.00,0.00) | 1.98(1.76,2.20) | 0.05(0.04,0.08) | 0.09(0.06,0.14) | 2.05(1.84,2.26) |
| Dominican Republic | 0.00(0.00,0.00) | 0.00(0.00,0.00) | 0.62(0.45,0.80) | 0.11(0.07,0.15) | 0.12(0.07,0.19) | 0.62(0.44,0.81) |
| Ecuador | 0.00(0.00,0.00) | 0.00(0.00,0.00) | 0.07(-0.55,0.69) | 0.03(0.02,0.05) | 0.03(0.02,0.05) | -0.09(-0.73,0.55) |
| Egypt | 0.00(0.00,0.00) | 0.00(0.00,0.00) | -0.82(-1.12,-0.51) | 0.02(0.01,0.03) | 0.02(0.01,0.02) | -0.91(-1.19,-0.64) |
| El Salvador | 0.00(0.00,0.00) | 0.00(0.00,0.00) | 1.13(0.97,1.28) | 0.05(0.03,0.07) | 0.07(0.04,0.10) | 1.12(0.96,1.28) |
| Equatorial Guinea | 0.00(0.00,0.00) | 0.00(0.00,0.00) | 0.30(0.17,0.44) | 0.05(0.03,0.09) | 0.06(0.03,0.11) | 0.38(0.25,0.51) |
| Eritrea | 0.01(0.01,0.01) | 0.01(0.01,0.02) | 0.62(0.55,0.69) | 0.35(0.22,0.53) | 0.41(0.22,0.67) | 0.62(0.55,0.68) |
| Estonia | 0.00(0.00,0.00) | 0.00(0.00,0.00) | -4.80(-4.98,-4.62) | 0.06(0.04,0.09) | 0.01(0.01,0.02) | -4.95(-5.14,-4.77) |
| Eswatini | 0.00(0.00,0.00) | 0.00(0.00,0.00) | -0.09(-0.71,0.52) | 0.10(0.06,0.15) | 0.10(0.05,0.16) | 0.23(-0.40,0.85) |
| Ethiopia | 0.01(0.00,0.01) | 0.01(0.00,0.01) | -0.79(-1.10,-0.48) | 0.33(0.19,0.49) | 0.29(0.17,0.49) | -0.86(-1.17,-0.55) |
| Fiji | 0.00(0.00,0.00) | 0.00(0.00,0.00) | -0.27(-0.53,-0.01) | 0.05(0.03,0.08) | 0.05(0.03,0.07) | -0.31(-0.57,-0.04) |
| Finland | 0.00(0.00,0.00) | 0.00(0.00,0.00) | -2.64(-2.89,-2.38) | 0.01(0.01,0.02) | 0.01(0.00,0.01) | -2.69(-2.96,-2.43) |
| France | 0.00(0.00,0.00) | 0.00(0.00,0.00) | -4.07(-4.19,-3.95) | 0.07(0.05,0.11) | 0.02(0.01,0.03) | -4.07(-4.18,-3.95) |
| Gabon | 0.00(0.00,0.00) | 0.00(0.00,0.00) | -1.06(-1.28,-0.85) | 0.07(0.04,0.11) | 0.05(0.03,0.09) | -1.09(-1.31,-0.87) |
| Gambia | 0.00(0.00,0.00) | 0.00(0.00,0.00) | 0.92(0.71,1.14) | 0.03(0.02,0.05) | 0.04(0.03,0.07) | 0.97(0.75,1.20) |
| Georgia | 0.00(0.00,0.00) | 0.00(0.00,0.00) | 0.92(0.37,1.48) | 0.08(0.05,0.12) | 0.10(0.06,0.15) | 0.79(0.25,1.34) |
| Germany | 0.00(0.00,0.00) | 0.00(0.00,0.00) | -2.59(-2.98,-2.20) | 0.04(0.02,0.05) | 0.01(0.01,0.02) | -2.85(-3.21,-2.49) |
| Ghana | 0.00(0.00,0.00) | 0.00(0.00,0.00) | -10.89(-12.58,-9.16) | 0.05(0.02,0.08) | 0.00(0.00,0.01) | -10.86(-12.55,-9.14) |
| Greece | 0.00(0.00,0.00) | 0.00(0.00,0.00) | -1.69(-1.91,-1.48) | 0.03(0.02,0.04) | 0.02(0.01,0.03) | -1.83(-2.07,-1.59) |
| Greenland | 0.02(0.01,0.02) | 0.01(0.00,0.01) | -3.56(-3.67,-3.45) | 0.74(0.48,1.06) | 0.23(0.14,0.35) | -3.76(-3.87,-3.64) |
| Grenada | 0.00(0.00,0.00) | 0.00(0.00,0.01) | 1.35(1.05,1.65) | 0.11(0.07,0.15) | 0.14(0.09,0.21) | 1.20(0.91,1.48) |
| Guam | 0.01(0.01,0.01) | 0.01(0.00,0.01) | -0.82(-1.09,-0.56) | 0.40(0.24,0.61) | 0.28(0.15,0.46) | -0.71(-0.99,-0.42) |
| Guatemala | 0.00(0.00,0.00) | 0.00(0.00,0.00) | -1.39(-1.72,-1.05) | 0.09(0.06,0.11) | 0.06(0.04,0.09) | -1.47(-1.80,-1.13) |
| Guinea | 0.00(0.00,0.00) | 0.00(0.00,0.00) | 1.63(1.56,1.71) | 0.04(0.02,0.06) | 0.06(0.04,0.11) | 1.66(1.58,1.73) |
| Guinea-Bissau | 0.00(0.00,0.00) | 0.00(0.00,0.00) | 1.32(1.20,1.43) | 0.05(0.03,0.07) | 0.07(0.04,0.12) | 1.39(1.28,1.50) |
| Guyana | 0.00(0.00,0.00) | 0.00(0.00,0.00) | 2.11(1.78,2.44) | 0.04(0.03,0.06) | 0.08(0.05,0.13) | 2.17(1.83,2.52) |
| Haiti | 0.00(0.00,0.00) | 0.00(0.00,0.01) | 2.21(2.03,2.39) | 0.10(0.05,0.15) | 0.16(0.09,0.25) | 2.19(2.01,2.37) |
| Honduras | 0.00(0.00,0.00) | 0.00(0.00,0.00) | 0.65(0.48,0.81) | 0.05(0.03,0.07) | 0.06(0.04,0.09) | 0.19(-0.01,0.39) |
| Hungary | 0.00(0.00,0.00) | 0.00(0.00,0.00) | -0.54(-0.83,-0.24) | 0.04(0.03,0.06) | 0.04(0.02,0.05) | -0.89(-1.19,-0.60) |
| Iceland | 0.00(0.00,0.00) | 0.00(0.00,0.00) | -2.23(-2.54,-1.91) | 0.03(0.02,0.04) | 0.02(0.01,0.02) | -2.14(-2.48,-1.80) |
| India | 0.01(0.00,0.01) | 0.01(0.00,0.01) | -1.28(-1.43,-1.14) | 0.32(0.20,0.47) | 0.23(0.14,0.34) | -1.36(-1.49,-1.23) |
| Indonesia | 0.01(0.01,0.01) | 0.01(0.01,0.02) | -0.20(-0.26,-0.13) | 0.43(0.27,0.63) | 0.40(0.23,0.64) | -0.34(-0.41,-0.27) |
| Iran (Islamic Republic of) | 0.00(0.00,0.00) | 0.00(0.00,0.00) | -0.95(-1.34,-0.56) | 0.03(0.02,0.05) | 0.03(0.02,0.03) | -0.89(-1.27,-0.50) |
| Iraq | 0.00(0.00,0.00) | 0.00(0.00,0.00) | -1.44(-1.71,-1.16) | 0.10(0.05,0.15) | 0.07(0.04,0.11) | -1.50(-1.78,-1.21) |
| Ireland | 0.00(0.00,0.00) | 0.00(0.00,0.00) | -2.28(-2.46,-2.09) | 0.02(0.01,0.03) | 0.01(0.01,0.02) | -2.17(-2.36,-1.97) |
| Israel | 0.00(0.00,0.00) | 0.00(0.00,0.00) | -1.55(-1.82,-1.27) | 0.02(0.01,0.03) | 0.01(0.01,0.02) | -1.60(-1.91,-1.29) |
| Italy | 0.00(0.00,0.00) | 0.00(0.00,0.00) | -3.06(-3.21,-2.91) | 0.04(0.03,0.06) | 0.02(0.01,0.02) | -3.18(-3.35,-3.02) |
| Jamaica | 0.00(0.00,0.00) | 0.00(0.00,0.00) | 1.40(0.83,1.97) | 0.06(0.04,0.08) | 0.10(0.06,0.17) | 1.35(0.73,1.97) |
| Japan | 0.00(0.00,0.00) | 0.00(0.00,0.00) | -0.94(-1.35,-0.52) | 0.02(0.01,0.03) | 0.02(0.01,0.02) | -1.15(-1.56,-0.73) |
| Jordan | 0.00(0.00,0.01) | 0.00(0.00,0.00) | -2.76(-3.02,-2.50) | 0.17(0.10,0.27) | 0.09(0.05,0.15) | -2.70(-2.94,-2.47) |
| Kazakhstan | 0.00(0.00,0.00) | 0.00(0.00,0.00) | 0.08(-0.16,0.31) | 0.10(0.07,0.14) | 0.11(0.07,0.15) | 0.02(-0.21,0.26) |
| Kenya | 0.01(0.00,0.01) | 0.01(0.01,0.02) | 2.06(1.96,2.16) | 0.36(0.20,0.59) | 0.61(0.35,0.96) | 1.97(1.87,2.07) |
| Kiribati | 0.00(0.00,0.00) | 0.00(0.00,0.00) | 2.73(2.28,3.18) | 0.07(0.04,0.11) | 0.12(0.06,0.21) | 2.75(2.31,3.20) |
| Kuwait | 0.00(0.00,0.00) | 0.00(0.00,0.00) | -2.62(-3.10,-2.13) | 0.14(0.09,0.21) | 0.05(0.03,0.08) | -2.67(-3.13,-2.21) |
| Kyrgyzstan | 0.00(0.00,0.00) | 0.00(0.00,0.00) | 0.55(0.17,0.92) | 0.12(0.07,0.19) | 0.12(0.07,0.18) | 0.56(0.20,0.92) |
| Lao People's Democratic Republic | 0.01(0.01,0.02) | 0.01(0.01,0.02) | -0.79(-1.01,-0.57) | 0.56(0.30,0.86) | 0.47(0.28,0.73) | -0.86(-1.08,-0.64) |
| Latvia | 0.00(0.00,0.00) | 0.00(0.00,0.00) | -4.75(-5.17,-4.34) | 0.05(0.03,0.07) | 0.01(0.01,0.02) | -4.94(-5.36,-4.51) |
| Lebanon | 0.00(0.00,0.00) | 0.00(0.00,0.00) | -1.26(-1.39,-1.12) | 0.12(0.06,0.21) | 0.08(0.04,0.12) | -1.25(-1.38,-1.12) |
| Lesotho | 0.00(0.00,0.00) | 0.00(0.00,0.00) | 2.29(1.61,2.97) | 0.07(0.04,0.12) | 0.12(0.07,0.19) | 2.63(1.96,3.32) |
| Liberia | 0.00(0.00,0.00) | 0.00(0.00,0.00) | 1.77(1.55,1.99) | 0.03(0.02,0.06) | 0.06(0.03,0.10) | 1.91(1.69,2.13) |
| Libya | 0.01(0.01,0.02) | 0.01(0.01,0.02) | -0.69(-0.87,-0.52) | 0.59(0.33,0.97) | 0.50(0.29,0.81) | -0.66(-0.82,-0.49) |
| Lithuania | 0.00(0.00,0.00) | 0.00(0.00,0.00) | -3.38(-3.75,-3.00) | 0.04(0.02,0.06) | 0.01(0.01,0.02) | -3.49(-3.88,-3.10) |
| Luxembourg | 0.00(0.00,0.00) | 0.00(0.00,0.00) | -4.12(-4.27,-3.97) | 0.04(0.03,0.06) | 0.01(0.01,0.02) | -4.39(-4.54,-4.24) |
| Madagascar | 0.01(0.00,0.01) | 0.01(0.00,0.01) | 0.06(-0.04,0.17) | 0.32(0.20,0.47) | 0.33(0.18,0.52) | 0.04(-0.06,0.14) |
| Malawi | 0.00(0.00,0.00) | 0.00(0.00,0.00) | -0.61(-0.81,-0.41) | 0.14(0.09,0.22) | 0.13(0.07,0.22) | -0.65(-0.85,-0.46) |
| Malaysia | 0.06(0.04,0.08) | 0.04(0.03,0.07) | -1.65(-2.16,-1.13) | 2.55(1.59,3.74) | 1.93(1.17,2.86) | -1.69(-2.22,-1.15) |
| Maldives | 0.00(0.00,0.00) | 0.00(0.00,0.00) | -3.68(-3.92,-3.44) | 0.12(0.06,0.20) | 0.05(0.03,0.07) | -3.70(-3.94,-3.47) |
| Mali | 0.00(0.00,0.00) | 0.00(0.00,0.00) | 0.60(0.38,0.81) | 0.03(0.02,0.04) | 0.03(0.02,0.05) | 0.60(0.38,0.81) |
| Malta | 0.00(0.00,0.00) | 0.00(0.00,0.00) | -1.69(-1.87,-1.51) | 0.08(0.05,0.11) | 0.05(0.03,0.07) | -1.62(-1.81,-1.43) |
| Marshall Islands | 0.00(0.00,0.01) | 0.00(0.00,0.01) | -0.01(-0.10,0.08) | 0.15(0.09,0.24) | 0.15(0.08,0.25) | -0.01(-0.10,0.08) |
| Mauritania | 0.00(0.00,0.00) | 0.00(0.00,0.00) | 0.66(0.60,0.72) | 0.02(0.01,0.04) | 0.03(0.01,0.05) | 0.72(0.66,0.78) |
| Mauritius | 0.01(0.00,0.01) | 0.01(0.00,0.01) | -0.48(-0.72,-0.24) | 0.23(0.14,0.32) | 0.22(0.14,0.32) | -0.42(-0.67,-0.17) |
| Mexico | 0.00(0.00,0.00) | 0.00(0.00,0.00) | -0.83(-0.97,-0.69) | 0.05(0.03,0.07) | 0.04(0.03,0.06) | -0.78(-0.92,-0.64) |
| Micronesia (Federated States of) | 0.01(0.00,0.01) | 0.00(0.00,0.01) | -0.62(-0.72,-0.52) | 0.23(0.12,0.37) | 0.19(0.10,0.31) | -0.63(-0.72,-0.54) |
| Monaco | 0.00(0.00,0.00) | 0.00(0.00,0.00) | -0.88(-1.01,-0.75) | 0.02(0.01,0.03) | 0.01(0.01,0.02) | -0.81(-0.96,-0.66) |
| Mongolia | 0.00(0.00,0.00) | 0.00(0.00,0.00) | 0.92(0.74,1.10) | 0.07(0.04,0.12) | 0.10(0.05,0.16) | 0.97(0.78,1.16) |
| Montenegro | 0.00(0.00,0.00) | 0.00(0.00,0.00) | 0.12(-0.03,0.28) | 0.00(0.00,0.01) | 0.00(0.00,0.01) | -0.01(-0.18,0.15) |
| Morocco | 0.01(0.00,0.01) | 0.01(0.00,0.01) | -1.13(-1.19,-1.07) | 0.36(0.21,0.55) | 0.25(0.14,0.43) | -1.33(-1.41,-1.24) |
| Mozambique | 0.00(0.00,0.00) | 0.00(0.00,0.00) | 2.55(2.34,2.76) | 0.01(0.01,0.02) | 0.02(0.01,0.03) | 2.57(2.36,2.78) |
| Myanmar | 0.01(0.00,0.01) | 0.01(0.00,0.01) | 0.66(0.36,0.97) | 0.27(0.16,0.41) | 0.32(0.20,0.51) | 0.52(0.21,0.83) |
| Namibia | 0.00(0.00,0.00) | 0.00(0.00,0.00) | -1.29(-1.59,-0.98) | 0.10(0.06,0.16) | 0.08(0.04,0.12) | -1.09(-1.40,-0.79) |
| Nauru | 0.01(0.00,0.01) | 0.01(0.00,0.01) | -0.29(-0.58,-0.01) | 0.24(0.12,0.42) | 0.22(0.11,0.41) | -0.25(-0.53,0.03) |
| Nepal | 0.01(0.00,0.01) | 0.00(0.00,0.01) | -1.01(-1.21,-0.80) | 0.30(0.18,0.46) | 0.21(0.12,0.33) | -1.10(-1.29,-0.90) |
| Netherlands | 0.00(0.00,0.00) | 0.00(0.00,0.00) | -1.39(-1.67,-1.10) | 0.02(0.02,0.03) | 0.02(0.01,0.02) | -1.47(-1.76,-1.19) |
| New Zealand | 0.00(0.00,0.00) | 0.00(0.00,0.00) | -2.19(-2.39,-1.99) | 0.03(0.02,0.04) | 0.01(0.01,0.02) | -2.17(-2.37,-1.97) |
| Nicaragua | 0.00(0.00,0.00) | 0.00(0.00,0.00) | 1.73(1.35,2.12) | 0.03(0.02,0.05) | 0.05(0.03,0.07) | 1.58(1.22,1.94) |
| Niger | 0.00(0.00,0.00) | 0.00(0.00,0.00) | 0.72(0.44,1.01) | 0.03(0.02,0.05) | 0.04(0.02,0.07) | 0.78(0.50,1.07) |
| Nigeria | 0.00(0.00,0.01) | 0.00(0.00,0.01) | -0.43(-0.67,-0.19) | 0.20(0.12,0.29) | 0.19(0.10,0.30) | -0.40(-0.64,-0.15) |
| Niue | 0.00(0.00,0.01) | 0.00(0.00,0.01) | -0.64(-0.76,-0.52) | 0.16(0.09,0.25) | 0.14(0.08,0.22) | -0.65(-0.79,-0.52) |
| North Macedonia | 0.00(0.00,0.00) | 0.00(0.00,0.00) | 0.96(0.77,1.15) | 0.01(0.01,0.02) | 0.01(0.01,0.02) | 0.77(0.57,0.97) |
| Northern Mariana Islands | 0.01(0.00,0.01) | 0.01(0.00,0.01) | 0.24(-0.21,0.68) | 0.31(0.18,0.53) | 0.33(0.20,0.52) | 0.12(-0.36,0.61) |
| Norway | 0.00(0.00,0.00) | 0.00(0.00,0.00) | -3.39(-3.58,-3.20) | 0.01(0.01,0.02) | 0.00(0.00,0.01) | -3.43(-3.63,-3.23) |
| Oman | 0.00(0.00,0.00) | 0.00(0.00,0.00) | -0.47(-0.71,-0.23) | 0.07(0.04,0.12) | 0.05(0.03,0.09) | -0.44(-0.67,-0.21) |
| Pakistan | 0.01(0.00,0.01) | 0.01(0.01,0.01) | 1.33(1.09,1.57) | 0.26(0.15,0.42) | 0.41(0.26,0.60) | 1.35(1.11,1.60) |
| Palau | 0.00(0.00,0.00) | 0.00(0.00,0.00) | 0.00(-0.29,0.29) | 0.01(0.00,0.02) | 0.01(0.00,0.01) | 0.05(-0.25,0.35) |
| Palestine | 0.00(0.00,0.00) | 0.00(0.00,0.00) | -1.42(-1.60,-1.25) | 0.05(0.03,0.08) | 0.03(0.02,0.05) | -1.47(-1.64,-1.30) |
| Panama | 0.00(0.00,0.00) | 0.00(0.00,0.00) | 0.63(0.40,0.87) | 0.05(0.04,0.07) | 0.07(0.04,0.10) | 0.66(0.41,0.92) |
| Papua New Guinea | 0.00(0.00,0.00) | 0.00(0.00,0.00) | 0.13(-0.00,0.26) | 0.10(0.05,0.18) | 0.11(0.05,0.19) | 0.12(-0.01,0.24) |
| Paraguay | 0.00(0.00,0.00) | 0.00(0.00,0.00) | 2.37(2.03,2.71) | 0.03(0.02,0.05) | 0.06(0.04,0.10) | 2.25(1.90,2.60) |
| Peru | 0.00(0.00,0.00) | 0.00(0.00,0.00) | -0.33(-0.50,-0.15) | 0.03(0.02,0.05) | 0.03(0.02,0.04) | -0.48(-0.66,-0.30) |
| Philippines | 0.01(0.01,0.01) | 0.01(0.01,0.02) | -0.01(-0.07,0.05) | 0.49(0.31,0.68) | 0.46(0.31,0.66) | -0.18(-0.24,-0.13) |
| Poland | 0.00(0.00,0.00) | 0.00(0.00,0.00) | -0.26(-0.56,0.04) | 0.02(0.02,0.03) | 0.02(0.01,0.03) | -0.43(-0.72,-0.14) |
| Portugal | 0.00(0.00,0.00) | 0.00(0.00,0.00) | -2.78(-3.01,-2.56) | 0.05(0.03,0.08) | 0.02(0.02,0.04) | -3.01(-3.26,-2.77) |
| Puerto Rico | 0.00(0.00,0.00) | 0.00(0.00,0.00) | -1.61(-1.89,-1.32) | 0.02(0.01,0.03) | 0.01(0.01,0.02) | -1.63(-1.91,-1.36) |
| Qatar | 0.00(0.00,0.00) | 0.00(0.00,0.00) | -2.09(-2.72,-1.46) | 0.09(0.05,0.15) | 0.05(0.03,0.09) | -2.04(-2.64,-1.44) |
| Republic of Korea | 0.00(0.00,0.00) | 0.00(0.00,0.00) | -2.67(-2.83,-2.50) | 0.04(0.03,0.06) | 0.02(0.01,0.03) | -2.79(-2.95,-2.64) |
| Republic of Moldova | 0.00(0.00,0.00) | 0.00(0.00,0.00) | -1.94(-2.18,-1.70) | 0.03(0.02,0.05) | 0.02(0.01,0.03) | -2.06(-2.30,-1.82) |
| Romania | 0.00(0.00,0.00) | 0.00(0.00,0.00) | 0.69(0.22,1.15) | 0.04(0.02,0.05) | 0.05(0.03,0.08) | 0.42(-0.03,0.87) |
| Russian Federation | 0.00(0.00,0.00) | 0.00(0.00,0.00) | -1.05(-1.36,-0.74) | 0.02(0.01,0.03) | 0.02(0.01,0.02) | -1.02(-1.35,-0.69) |
| Rwanda | 0.01(0.01,0.01) | 0.01(0.00,0.01) | -2.15(-2.41,-1.89) | 0.42(0.26,0.63) | 0.26(0.14,0.44) | -2.26(-2.52,-1.99) |
| Saint Kitts and Nevis | 0.00(0.00,0.01) | 0.00(0.00,0.01) | -0.87(-1.28,-0.47) | 0.20(0.14,0.28) | 0.13(0.08,0.19) | -1.36(-1.82,-0.89) |
| Saint Lucia | 0.00(0.00,0.00) | 0.00(0.00,0.01) | -0.26(-0.34,-0.18) | 0.16(0.11,0.21) | 0.15(0.10,0.22) | -0.26(-0.34,-0.18) |
| Saint Vincent and the Grenadines | 0.00(0.00,0.00) | 0.00(0.00,0.00) | 1.21(1.06,1.36) | 0.09(0.06,0.13) | 0.14(0.09,0.20) | 1.14(0.97,1.31) |
| Samoa | 0.01(0.00,0.01) | 0.01(0.00,0.01) | -0.87(-1.08,-0.66) | 0.31(0.19,0.47) | 0.27(0.16,0.45) | -0.83(-1.04,-0.61) |
| San Marino | 0.00(0.00,0.00) | 0.00(0.00,0.00) | -1.89(-2.16,-1.62) | 0.06(0.04,0.09) | 0.03(0.01,0.05) | -1.89(-2.16,-1.62) |
| Sao Tome and Principe | 0.00(0.00,0.00) | 0.00(0.00,0.00) | 0.14(-0.19,0.47) | 0.00(0.00,0.01) | 0.01(0.00,0.01) | 0.14(-0.20,0.48) |
| Saudi Arabia | 0.01(0.00,0.01) | 0.01(0.00,0.01) | -1.78(-2.03,-1.53) | 0.35(0.19,0.57) | 0.22(0.13,0.37) | -1.73(-1.98,-1.47) |
| Senegal | 0.00(0.00,0.00) | 0.00(0.00,0.00) | 0.13(-0.01,0.27) | 0.04(0.02,0.06) | 0.04(0.02,0.07) | 0.23(0.08,0.37) |
| Serbia | 0.00(0.00,0.00) | 0.00(0.00,0.00) | -0.38(-0.55,-0.22) | 0.02(0.01,0.04) | 0.02(0.01,0.03) | -0.61(-0.77,-0.45) |
| Seychelles | 0.01(0.01,0.02) | 0.01(0.01,0.01) | -0.17(-0.46,0.11) | 0.45(0.27,0.67) | 0.42(0.26,0.65) | -0.20(-0.48,0.08) |
| Sierra Leone | 0.00(0.00,0.00) | 0.00(0.00,0.00) | 1.44(1.34,1.54) | 0.02(0.01,0.04) | 0.04(0.02,0.06) | 1.56(1.45,1.67) |
| Singapore | 0.01(0.01,0.02) | 0.00(0.00,0.00) | -4.23(-4.43,-4.03) | 0.50(0.32,0.71) | 0.12(0.08,0.18) | -4.54(-4.74,-4.35) |
| Slovakia | 0.00(0.00,0.00) | 0.00(0.00,0.00) | -1.21(-1.56,-0.86) | 0.06(0.03,0.09) | 0.03(0.02,0.06) | -1.34(-1.68,-1.00) |
| Slovenia | 0.00(0.00,0.00) | 0.00(0.00,0.00) | -4.24(-4.99,-3.49) | 0.03(0.02,0.05) | 0.01(0.01,0.02) | -4.37(-5.13,-3.62) |
| Solomon Islands | 0.00(0.00,0.01) | 0.01(0.00,0.01) | 4.03(3.63,4.42) | 0.12(0.06,0.21) | 0.30(0.16,0.51) | 4.09(3.69,4.49) |
| Somalia | 0.01(0.00,0.01) | 0.01(0.00,0.01) | 0.06(-0.00,0.12) | 0.28(0.15,0.45) | 0.29(0.15,0.49) | 0.01(-0.06,0.08) |
| South Africa | 0.00(0.00,0.00) | 0.00(0.00,0.00) | -3.24(-3.48,-3.00) | 0.12(0.08,0.17) | 0.05(0.03,0.07) | -3.22(-3.48,-2.96) |
| South Sudan | 0.01(0.00,0.01) | 0.01(0.00,0.01) | 0.29(0.00,0.59) | 0.22(0.12,0.38) | 0.25(0.13,0.44) | 0.31(0.00,0.61) |
| Spain | 0.00(0.00,0.00) | 0.00(0.00,0.00) | -3.80(-4.11,-3.50) | 0.05(0.03,0.07) | 0.02(0.01,0.02) | -4.03(-4.35,-3.71) |
| Sri Lanka | 0.00(0.00,0.01) | 0.00(0.00,0.01) | 0.10(-0.21,0.41) | 0.17(0.11,0.26) | 0.18(0.10,0.30) | -0.08(-0.40,0.24) |
| Sudan | 0.00(0.00,0.00) | 0.00(0.00,0.00) | -1.17(-1.28,-1.07) | 0.09(0.04,0.15) | 0.06(0.03,0.11) | -1.16(-1.25,-1.06) |
| Suriname | 0.00(0.00,0.00) | 0.00(0.00,0.01) | 1.62(1.33,1.91) | 0.12(0.08,0.16) | 0.17(0.11,0.26) | 1.58(1.30,1.86) |
| Sweden | 0.00(0.00,0.00) | 0.00(0.00,0.00) | -2.12(-2.34,-1.91) | 0.01(0.01,0.02) | 0.01(0.00,0.01) | -2.14(-2.36,-1.93) |
| Switzerland | 0.00(0.00,0.00) | 0.00(0.00,0.00) | -5.49(-6.12,-4.86) | 0.06(0.04,0.08) | 0.01(0.01,0.02) | -5.76(-6.36,-5.16) |
| Syrian Arab Republic | 0.00(0.00,0.00) | 0.00(0.00,0.00) | -3.30(-3.75,-2.84) | 0.04(0.02,0.06) | 0.02(0.01,0.03) | -3.37(-3.82,-2.93) |
| Taiwan (Province of China) | 0.05(0.03,0.07) | 0.02(0.01,0.03) | -2.94(-3.09,-2.79) | 2.31(1.44,3.27) | 0.98(0.60,1.47) | -3.05(-3.20,-2.90) |
| Tajikistan | 0.00(0.00,0.00) | 0.00(0.00,0.00) | -1.56(-1.81,-1.30) | 0.09(0.05,0.14) | 0.07(0.04,0.10) | -1.50(-1.83,-1.17) |
| Thailand | 0.01(0.01,0.02) | 0.01(0.01,0.02) | -0.43(-0.73,-0.14) | 0.47(0.30,0.69) | 0.50(0.30,0.75) | -0.40(-0.75,-0.05) |
| Timor-Leste | 0.01(0.00,0.01) | 0.01(0.00,0.01) | 0.29(-0.04,0.63) | 0.26(0.14,0.46) | 0.31(0.17,0.50) | 0.18(-0.19,0.55) |
| Togo | 0.00(0.00,0.00) | 0.00(0.00,0.00) | 1.21(1.11,1.32) | 0.06(0.03,0.09) | 0.08(0.05,0.14) | 1.24(1.14,1.34) |
| Tokelau | 0.00(0.00,0.01) | 0.00(0.00,0.01) | -0.58(-0.67,-0.49) | 0.18(0.09,0.30) | 0.16(0.09,0.26) | -0.53(-0.63,-0.42) |
| Tonga | 0.00(0.00,0.00) | 0.00(0.00,0.00) | -0.46(-0.64,-0.28) | 0.11(0.07,0.19) | 0.11(0.06,0.19) | -0.42(-0.61,-0.24) |
| Trinidad and Tobago | 0.00(0.00,0.00) | 0.00(0.00,0.00) | 1.23(0.87,1.60) | 0.07(0.05,0.09) | 0.11(0.07,0.16) | 1.35(0.96,1.73) |
| Tunisia | 0.01(0.01,0.02) | 0.01(0.01,0.02) | -0.84(-0.93,-0.75) | 0.58(0.36,0.86) | 0.49(0.27,0.76) | -0.78(-0.88,-0.69) |
| Turkmenistan | 0.00(0.00,0.00) | 0.00(0.00,0.00) | 1.50(0.88,2.12) | 0.28(0.16,0.43) | 0.13(0.08,0.20) | -2.66(-2.89,-2.43) |
| Tuvalu | 0.00(0.00,0.01) | 0.00(0.00,0.01) | -0.34(-0.41,-0.26) | 0.07(0.05,0.10) | 0.11(0.07,0.16) | 1.58(0.97,2.19) |
| T眉rkiye | 0.01(0.00,0.01) | 0.00(0.00,0.00) | -2.56(-2.79,-2.33) | 0.20(0.12,0.33) | 0.18(0.11,0.30) | -0.35(-0.43,-0.28) |
| Uganda | 0.01(0.01,0.02) | 0.02(0.01,0.03) | -0.61(-1.05,-0.16) | 0.61(0.37,0.94) | 0.71(0.42,1.16) | -0.60(-1.06,-0.14) |
| Ukraine | 0.00(0.00,0.00) | 0.00(0.00,0.00) | 0.93(0.37,1.50) | 0.01(0.01,0.02) | 0.02(0.01,0.04) | 0.96(0.40,1.52) |
| United Arab Emirates | 0.01(0.00,0.01) | 0.00(0.00,0.00) | -1.82(-2.01,-1.63) | 0.22(0.11,0.41) | 0.11(0.07,0.17) | -1.93(-2.10,-1.75) |
| United Kingdom | 0.00(0.00,0.00) | 0.00(0.00,0.00) | -1.12(-1.23,-1.00) | 0.02(0.02,0.03) | 0.02(0.01,0.02) | -1.06(-1.17,-0.95) |
| United Republic of Tanzania | 0.01(0.00,0.01) | 0.01(0.00,0.01) | 0.30(0.22,0.37) | 0.34(0.20,0.54) | 0.37(0.21,0.60) | 0.26(0.19,0.34) |
| United States of America | 0.00(0.00,0.00) | 0.00(0.00,0.00) | -1.97(-2.08,-1.86) | 0.03(0.02,0.04) | 0.02(0.01,0.02) | -1.94(-2.05,-1.82) |
| United States Virgin Islands | 0.00(0.00,0.00) | 0.00(0.00,0.00) | -0.27(-0.45,-0.08) | 0.15(0.10,0.21) | 0.13(0.07,0.20) | 0.14(-0.04,0.32) |
| Uruguay | 0.00(0.00,0.01) | 0.00(0.00,0.00) | -1.19(-1.46,-0.91) | 0.15(0.10,0.23) | 0.10(0.06,0.15) | -1.12(-1.40,-0.84) |
| Uzbekistan | 0.00(0.00,0.00) | 0.00(0.00,0.01) | 2.70(2.46,2.95) | 0.08(0.04,0.11) | 0.15(0.09,0.23) | 2.73(2.48,2.98) |
| Vanuatu | 0.00(0.00,0.01) | 0.00(0.00,0.01) | -0.15(-0.27,-0.03) | 0.16(0.09,0.26) | 0.16(0.09,0.26) | -0.15(-0.27,-0.03) |
| Venezuela (Bolivarian Republic of) | 0.00(0.00,0.00) | 0.00(0.00,0.00) | 0.59(0.27,0.92) | 0.06(0.04,0.08) | 0.08(0.05,0.12) | 0.76(0.44,1.07) |
| Viet Nam | 0.01(0.01,0.02) | 0.02(0.01,0.03) | 1.66(1.45,1.86) | 0.64(0.41,0.93) | 0.91(0.54,1.40) | 1.57(1.37,1.78) |
| Yemen | 0.00(0.00,0.00) | 0.00(0.00,0.00) | -0.87(-0.98,-0.76) | 0.06(0.03,0.10) | 0.05(0.03,0.08) | -0.84(-0.96,-0.73) |
| Zambia | 0.01(0.01,0.01) | 0.02(0.01,0.03) | 1.70(1.09,2.31) | 0.46(0.29,0.67) | 0.70(0.23,1.45) | 1.66(1.05,2.28) |
| Zimbabwe | 0.00(0.00,0.00) | 0.00(0.00,0.01) | 1.09(0.44,1.75) | 0.11(0.07,0.17) | 0.15(0.09,0.24) | 1.52(0.83,2.22) |

**Table S2:** Frontier analysis of deaths rate from 2022 to 2025 (both male and female).

| sex | val | Time | group | low_95 | up_95 |
| --- | --- | --- | --- | --- | --- |
| both |  |  |  |  |  |
|  | 0.01031821443392174 | 1990 | ASR | 0.009695983841834472 | 0.01094044502600901 |
|  | 0.0103169923164168 | 1991 | ASR | 0.009773643584958532 | 0.01086034104787506 |
|  | 0.01029978227520028 | 1992 | ASR | 0.009789538468846077 | 0.01081002608155448 |
|  | 0.01027464451641849 | 1993 | ASR | 0.009779894638693705 | 0.01076939439414327 |
|  | 0.01021305625426316 | 1994 | ASR | 0.009727871177714722 | 0.0106982413308116 |
|  | 0.01013537131126587 | 1995 | ASR | 0.009656245685072989 | 0.01061449693745875 |
|  | 0.01003121771221477 | 1996 | ASR | 0.009557759479733792 | 0.01050467594469575 |
|  | 0.009912571048242226 | 1997 | ASR | 0.009445667074658232 | 0.01037947502182622 |
|  | 0.009777113787724617 | 1998 | ASR | 0.0093180599325374 | 0.01023616764291183 |
|  | 0.009597448296825127 | 1999 | ASR | 0.009147456240791826 | 0.01004744035285843 |
|  | 0.009376846462326735 | 2000 | ASR | 0.008936182109815137 | 0.009817510814838334 |
|  | 0.009098534913746953 | 2001 | ASR | 0.00866938157736559 | 0.009527688250128317 |
|  | 0.008798966460278225 | 2002 | ASR | 0.008383011729226082 | 0.009214921191330369 |
|  | 0.008487680133631334 | 2003 | ASR | 0.008086284559103582 | 0.008889075708159087 |
|  | 0.008186965903256 | 2004 | ASR | 0.007798766092684567 | 0.008575165713827434 |
|  | 0.00792862561861029 | 2005 | ASR | 0.007550150055139489 | 0.00830710118208109 |
|  | 0.007709063290751931 | 2006 | ASR | 0.007338100762325553 | 0.00808002581917831 |
|  | 0.007537781887990434 | 2007 | ASR | 0.007174158778582073 | 0.007901404997398795 |
|  | 0.007391168687697653 | 2008 | ASR | 0.007035913959414902 | 0.007746423415980404 |
|  | 0.007265231316730571 | 2009 | ASR | 0.006918044823375297 | 0.007612417810085845 |
|  | 0.00716705537933262 | 2010 | ASR | 0.006825955582362446 | 0.007508155176302794 |
|  | 0.007073456675447855 | 2011 | ASR | 0.006736812303054713 | 0.007410101047840997 |
|  | 0.006980390072107854 | 2012 | ASR | 0.006647952134890767 | 0.007312828009324941 |
|  | 0.006913021458079034 | 2013 | ASR | 0.006585135155718839 | 0.007240907760439229 |
|  | 0.006865846163306278 | 2014 | ASR | 0.006542368963460925 | 0.007189323363151631 |
|  | 0.006827850649617603 | 2015 | ASR | 0.006507680168916619 | 0.007148021130318587 |
|  | 0.006816092349565689 | 2016 | ASR | 0.0064973597506266 | 0.007134824948504778 |
|  | 0.006810845002851806 | 2017 | ASR | 0.006492442560114376 | 0.007129247445589236 |
|  | 0.006795559402489475 | 2018 | ASR | 0.006476720136666113 | 0.007114398668312836 |
|  | 0.00678652389784948 | 2019 | ASR | 0.006462607682972251 | 0.007110440112726708 |
|  | 0.006756127956126868 | 2020 | ASR | 0.006415980989069526 | 0.007096274923184211 |
|  | 0.006696037632910921 | 2021 | ASR | 0.006309704486945636 | 0.007082370778876206 |
|  | 0.006552094830316909 | 2022 | ASR | 0.006024357847639393 | 0.007079831812994426 |
|  | 0.006424104531241855 | 2023 | ASR | 0.005799977533051289 | 0.007048231529432421 |
|  | 0.006298424215061425 | 2024 | ASR | 0.005592768346080674 | 0.007004080084042175 |
|  | 0.006174378465274698 | 2025 | ASR | 0.005396546116562017 | 0.00695221081398738 |
|  | 0.006051159839995713 | 2026 | ASR | 0.005207689207676905 | 0.006894630472314521 |
|  | 0.005928077002343987 | 2027 | ASR | 0.005024332743169801 | 0.006831821261518174 |
|  | 0.005805353719320579 | 2028 | ASR | 0.004846391398427157 | 0.006764316040214001 |
|  | 0.005683196386725953 | 2029 | ASR | 0.004673023214795523 | 0.006693369558656383 |
|  | 0.005561825407650946 | 2030 | ASR | 0.004503191703551564 | 0.006620459111750328 |
|  | 0.005441628921171835 | 2031 | ASR | 0.004336369272438553 | 0.006546888569905118 |
|  | 0.005322760512858983 | 2032 | ASR | 0.004172280006692067 | 0.006473241019025899 |
|  | 0.005205736583564695 | 2033 | ASR | 0.00401135943314156 | 0.00640011373398783 |
|  | 0.005090315310491247 | 2034 | ASR | 0.003852896194696824 | 0.006327734426285671 |
|  | 0.0049759676880929 | 2035 | ASR | 0.003695716052170893 | 0.006256219324014907 |
|  | 0.00486220411988615 | 2036 | ASR | 0.003538824405814217 | 0.006185583833958083 |
|  | 0.004748687064821096 | 2037 | ASR | 0.003381614318616206 | 0.006115759811025986 |
|  | 0.004635623601280332 | 2038 | ASR | 0.003224260157488328 | 0.006046987045072336 |
|  | 0.004523069080802251 | 2039 | ASR | 0.003066499611940239 | 0.005979638549664264 |
|  | 0.004410975849882612 | 2040 | ASR | 0.002907847436420498 | 0.005914104263344727 |
|  | 0.004299505406007398 | 2041 | ASR | 0.002748027753360778 | 0.005850983058654018 |
|  | 0.00418878264885774 | 2042 | ASR | 0.002586930485578993 | 0.005790634812136486 |
|  | 0.004079260758631467 | 2043 | ASR | 0.002424994394830334 | 0.0057335271224326 |
|  | 0.003971025548442288 | 2044 | ASR | 0.002262265679300574 | 0.005679785417584002 |
|  | 0.003864126618415893 | 2045 | ASR | 0.002098701898710536 | 0.005629551338121249 |
|  | 0.003758692081584005 | 2046 | ASR | 0.001934366782507124 | 0.005583017380660887 |
|  | 0.003654974895119814 | 2047 | ASR | 0.001769520971731902 | 0.005540428818507725 |
|  | 0.003553362366169797 | 2048 | ASR | 0.001604745264853755 | 0.00550197946748584 |
|  | 0.003453976236370562 | 2049 | ASR | 0.001440333972863918 | 0.005467618499877206 |
|  | 0.003356722045431606 | 2050 | ASR | 0.001276401428496269 | 0.005437042662366944 |
| male |  |  |  |  |  |
|  | 0.02064201331635844 | 1990 | ASR | 0.01914460007391324 | 0.02213942655880364 |
|  | 0.02060719704857781 | 1991 | ASR | 0.01927853800390546 | 0.02193585609325016 |
|  | 0.0205694878648693 | 1992 | ASR | 0.01932478041533106 | 0.02181419531440754 |
|  | 0.02048516610468551 | 1993 | ASR | 0.01928429635175721 | 0.02168603585761381 |
|  | 0.0203735706524057 | 1994 | ASR | 0.01919910841260843 | 0.02154803289220297 |
|  | 0.02023638394444868 | 1995 | ASR | 0.01907843493890201 | 0.02139433294999535 |
|  | 0.02005470458877866 | 1996 | ASR | 0.01891116635795229 | 0.02119824281960503 |
|  | 0.01983633585562665 | 1997 | ASR | 0.0187063404710919 | 0.0209663312401614 |
|  | 0.01957837981214199 | 1998 | ASR | 0.01846485805381469 | 0.02069190157046929 |
|  | 0.0192284906874666 | 1999 | ASR | 0.01813541926175087 | 0.02032156211318233 |
|  | 0.01884568269241512 | 2000 | ASR | 0.01777390417378456 | 0.01991746121104569 |
|  | 0.01835106513862422 | 2001 | ASR | 0.01730633509568149 | 0.01939579518156695 |
|  | 0.01783516906682529 | 2002 | ASR | 0.01681973444368186 | 0.01885060368996871 |
|  | 0.01728967606904426 | 2003 | ASR | 0.01630299167652898 | 0.01827636046155954 |
|  | 0.01676291339102888 | 2004 | ASR | 0.01580269933508144 | 0.01772312744697633 |
|  | 0.01628717898360276 | 2005 | ASR | 0.0153477723078671 | 0.01722658565933842 |
|  | 0.01588437725564159 | 2006 | ASR | 0.01496232529897893 | 0.01680642921230424 |
|  | 0.01559114850759822 | 2007 | ASR | 0.0146846968657185 | 0.01649760014947795 |
|  | 0.01530934735671593 | 2008 | ASR | 0.01441972157539874 | 0.01619897313803311 |
|  | 0.01507075754260883 | 2009 | ASR | 0.01419772971699442 | 0.01594378536822325 |
|  | 0.01488435227520312 | 2010 | ASR | 0.01402574426240797 | 0.01574296028799826 |
|  | 0.01472542864507021 | 2011 | ASR | 0.0138791035535695 | 0.01557175373657093 |
|  | 0.01457480249166707 | 2012 | ASR | 0.01373892625307857 | 0.01541067873025558 |
|  | 0.0144631773695638 | 2013 | ASR | 0.01363743426903325 | 0.01528892047009435 |
|  | 0.01436278752385849 | 2014 | ASR | 0.01354710574587361 | 0.01517846930184337 |
|  | 0.01429351916209103 | 2015 | ASR | 0.01348679864517309 | 0.01510023967900896 |
|  | 0.01425083879704534 | 2016 | ASR | 0.01344882873704631 | 0.01505284885704437 |
|  | 0.01421581168553278 | 2017 | ASR | 0.01341587952195305 | 0.01501574384911251 |
|  | 0.01417847794377569 | 2018 | ASR | 0.01337338271417356 | 0.01498357317337783 |
|  | 0.01411485533563241 | 2019 | ASR | 0.01329481052747926 | 0.01493490014378555 |
|  | 0.01404073510223402 | 2020 | ASR | 0.01317828105639832 | 0.01490318914806972 |
|  | 0.01390868215949438 | 2021 | ASR | 0.01294759293766608 | 0.01486977138132268 |
|  | 0.0136349544620842 | 2022 | ASR | 0.01242044765616487 | 0.01484946126800352 |
|  | 0.01339513716649098 | 2023 | ASR | 0.01200249715664582 | 0.01478777717633614 |
|  | 0.01315740199643029 | 2024 | ASR | 0.01160885591607735 | 0.01470594807678324 |
|  | 0.01292427895299863 | 2025 | ASR | 0.01123622067430275 | 0.01461233723169451 |
|  | 0.01269318285813622 | 2026 | ASR | 0.01087692297640271 | 0.01450944273986973 |
|  | 0.01246530735298975 | 2027 | ASR | 0.01052956507988505 | 0.01440104962609445 |
|  | 0.01223945658440329 | 2028 | ASR | 0.01019218122406618 | 0.0142867319447404 |
|  | 0.0120169695686276 | 2029 | ASR | 0.009863787721210101 | 0.0141701514160451 |
|  | 0.01179645965288203 | 2030 | ASR | 0.009541127252151652 | 0.0140517920536124 |
|  | 0.01157965737924459 | 2031 | ASR | 0.009224390668968041 | 0.01393492408952114 |
|  | 0.01136533762211108 | 2032 | ASR | 0.00891177073987078 | 0.01381890450435139 |
|  | 0.01115528323505391 | 2033 | ASR | 0.008604129523200521 | 0.01370643694690731 |
|  | 0.01094778994749275 | 2034 | ASR | 0.008299223315692663 | 0.01359635657929283 |
|  | 0.01074384931834257 | 2035 | ASR | 0.00799670698673213 | 0.01349099164995301 |
|  | 0.0105414757809786 | 2036 | ASR | 0.007694014494930009 | 0.0133889370670272 |
|  | 0.01034155831358786 | 2037 | ASR | 0.007391043519204649 | 0.01329207310797106 |
|  | 0.0101429473306767 | 2038 | ASR | 0.007086768373142966 | 0.01319912628821043 |
|  | 0.009946796591513188 | 2039 | ASR | 0.006781446431301061 | 0.01311214675172532 |
|  | 0.009751823653129233 | 2040 | ASR | 0.006473459936932026 | 0.01303018736932644 |
|  | 0.009559261813691149 | 2041 | ASR | 0.006163015166550599 | 0.0129555084608317 |
|  | 0.009368094845925493 | 2042 | ASR | 0.005849104333989932 | 0.01288708535786105 |
|  | 0.009179880540535713 | 2043 | ASR | 0.005532702524873648 | 0.01282705855619778 |
|  | 0.008993542110856257 | 2044 | ASR | 0.00521303283880059 | 0.01277405138291192 |
|  | 0.008810360464533908 | 2045 | ASR | 0.004890756320268207 | 0.01272996460879961 |
|  | 0.008629344595745918 | 2046 | ASR | 0.004565267989584917 | 0.01269342120190692 |
|  | 0.00845179587012149 | 2047 | ASR | 0.004237390012089004 | 0.01266620172815398 |
|  | 0.008277233545260753 | 2048 | ASR | 0.003907418890636184 | 0.01264704819988532 |
|  | 0.008106631921391737 | 2049 | ASR | 0.003575997274702784 | 0.01263726656808069 |
|  | 0.007938563169294909 | 2050 | ASR | 0.003242659073749155 | 0.01263446726484066 |
| female |  |  |  |  |  |
|  | 0.00819305015752585 | 1990 | ASR | 0.0074120466580961 | 0.0089740536569556 |
|  | 0.00810511118505325 | 1991 | ASR | 0.007405373663105542 | 0.008804848707000958 |
|  | 0.00801576218915273 | 1992 | ASR | 0.007369156066232154 | 0.008662368312073308 |
|  | 0.007926717385759645 | 1993 | ASR | 0.00731408835838821 | 0.008539346413131078 |
|  | 0.007823778391297855 | 1994 | ASR | 0.007234517562689432 | 0.008413039219906278 |
|  | 0.007712915566893708 | 1995 | ASR | 0.007141017981784428 | 0.008284813152002989 |
|  | 0.007585110151432387 | 1996 | ASR | 0.007029270880462389 | 0.008140949422402385 |
|  | 0.007455182288013487 | 1997 | ASR | 0.006912415041610574 | 0.007997949534416399 |
|  | 0.007310276881746153 | 1998 | ASR | 0.006780585754066079 | 0.007839968009426226 |
|  | 0.007155143772229169 | 1999 | ASR | 0.006638182692251592 | 0.007672104852206745 |
|  | 0.006977704260141924 | 2000 | ASR | 0.006475653290015284 | 0.007479755230268564 |
|  | 0.006775432296344627 | 2001 | ASR | 0.00628965091344751 | 0.007261213679241744 |
|  | 0.00656129701533343 | 2002 | ASR | 0.006091593417464048 | 0.007031000613202813 |
|  | 0.006349960547198103 | 2003 | ASR | 0.005893080803771768 | 0.006806840290624437 |
|  | 0.006147923341913046 | 2004 | ASR | 0.005700829232862852 | 0.006595017450963241 |
|  | 0.005960698370794206 | 2005 | ASR | 0.005521583469668709 | 0.006399813271919702 |
|  | 0.005786763416039375 | 2006 | ASR | 0.00535545742658932 | 0.006218069405489431 |
|  | 0.005635594060892611 | 2007 | ASR | 0.005212403980013844 | 0.006058784141771379 |
|  | 0.005491885390988532 | 2008 | ASR | 0.005077287309224361 | 0.005906483472752702 |
|  | 0.005370521044911488 | 2009 | ASR | 0.004965046524507775 | 0.005775995565315202 |
|  | 0.005258560119382093 | 2010 | ASR | 0.004862842700747539 | 0.005654277538016648 |
|  | 0.005156678752269606 | 2011 | ASR | 0.004770662778833023 | 0.00554269472570619 |
|  | 0.005060661387472081 | 2012 | ASR | 0.004683782404903482 | 0.005437540370040679 |
|  | 0.004975699505043583 | 2013 | ASR | 0.004607471821484526 | 0.00534392718860264 |
|  | 0.004900386506387516 | 2014 | ASR | 0.004538880777905178 | 0.005261892234869853 |
|  | 0.004828141760641043 | 2015 | ASR | 0.004471977087912721 | 0.005184306433369364 |
|  | 0.004765159898716271 | 2016 | ASR | 0.004410927080429563 | 0.005119392717002979 |
|  | 0.004710964938377567 | 2017 | ASR | 0.004354230199576854 | 0.005067699677178281 |
|  | 0.004649485054976847 | 2018 | ASR | 0.004284412200987926 | 0.005014557908965767 |
|  | 0.004588745248270324 | 2019 | ASR | 0.004208147480192019 | 0.00496934301634863 |
|  | 0.004518769797009226 | 2020 | ASR | 0.004114082487014168 | 0.004923457107004283 |
|  | 0.004438379213478594 | 2021 | ASR | 0.003995999458267952 | 0.004880758968689236 |
|  | 0.004329886097488906 | 2022 | ASR | 0.003823214472501934 | 0.004836557722475878 |
|  | 0.004233292361190737 | 2023 | ASR | 0.003676463586447486 | 0.004790121135933988 |
|  | 0.004138389706030881 | 2024 | ASR | 0.00353495151570674 | 0.004741827896355021 |
|  | 0.004047212327693708 | 2025 | ASR | 0.003400452516127363 | 0.004693972139260053 |
|  | 0.003958650611310266 | 2026 | ASR | 0.003271435569674608 | 0.004645865652945923 |
|  | 0.003873540316584101 | 2027 | ASR | 0.003147217878036492 | 0.00459986275513171 |
|  | 0.003790955526091142 | 2028 | ASR | 0.003026913734660828 | 0.004554997317521455 |
|  | 0.003711838113621175 | 2029 | ASR | 0.002910248714286709 | 0.00451342751295564 |
|  | 0.003634905285006709 | 2030 | ASR | 0.002795636540039754 | 0.004474174029973664 |
|  | 0.003561366976531071 | 2031 | ASR | 0.002683666094445671 | 0.004439067858616471 |
|  | 0.003490339226610657 | 2032 | ASR | 0.002573726994229867 | 0.004406951458991447 |
|  | 0.003422607054281598 | 2033 | ASR | 0.002465662048470628 | 0.004379552060092567 |
|  | 0.003356986234007574 | 2034 | ASR | 0.002358372437619949 | 0.0043556000303952 |
|  | 0.003294316452433585 | 2035 | ASR | 0.002252114983741211 | 0.004336517921125958 |
|  | 0.003233514082325384 | 2036 | ASR | 0.002146101951850151 | 0.004320926212800617 |
|  | 0.003175257576247365 | 2037 | ASR | 0.002040383704117039 | 0.004310131448377691 |
|  | 0.00311855974995321 | 2038 | ASR | 0.001934396816560438 | 0.004302722683345982 |
|  | 0.003064161768300801 | 2039 | ASR | 0.001828326280191962 | 0.00429999725640964 |
|  | 0.003011022660250967 | 2040 | ASR | 0.001721515433826284 | 0.004300529886675649 |
|  | 0.002959938877434455 | 2041 | ASR | 0.001614258981434954 | 0.004305618773433955 |
|  | 0.00291006382037762 | 2042 | ASR | 0.001506221517061928 | 0.004313906123693313 |
|  | 0.002862119843779105 | 2043 | ASR | 0.001397633235222446 | 0.004326606452335764 |
|  | 0.002815218007065471 | 2044 | ASR | 0.001288142468693243 | 0.004342293545437699 |
|  | 0.002770121583429398 | 2045 | ASR | 0.001178052932454223 | 0.004362190234404572 |
|  | 0.002725994719609466 | 2046 | ASR | 0.001067117980655164 | 0.004384871458563768 |
|  | 0.002683581244836531 | 2047 | ASR | 0.0009556192857675273 | 0.004411543203905535 |
|  | 0.002642249359422165 | 2048 | ASR | 0.0008435958175359479 | 0.004440902901308381 |
|  | 0.002602559936345475 | 2049 | ASR | 0.0007311184428954469 | 0.004474001429795503 |
|  | 0.002563436558229763 | 2050 | ASR | 0.000617976360287724 | 0.004508896756171802 |

**Table S3:** Frontier analysis of DALYs rate from 2022 to 2025 (both male and female).

| sex | val | Time | group | low_95 | up_95 |
| --- | --- | --- | --- | --- | --- |
| both |  |  |  |  |  |
|  | 0.4533387711550307 | 1990 | ASR | 0.4474658283097077 | 0.4592117140003538 |
|  | 0.4607310106782695 | 1991 | ASR | 0.4549901882293609 | 0.4664718331271782 |
|  | 0.4614928921327632 | 1992 | ASR | 0.4558130222497011 | 0.4671727620158254 |
|  | 0.4604137791032748 | 1993 | ASR | 0.4547998624587085 | 0.4660276957478411 |
|  | 0.4559993105906122 | 1994 | ASR | 0.4504738023450479 | 0.4615248188361765 |
|  | 0.4526752462201009 | 1995 | ASR | 0.4472248340686642 | 0.4581256583715375 |
|  | 0.4471252225712318 | 1996 | ASR | 0.4417638554879235 | 0.4524865896545401 |
|  | 0.442260583354312 | 1997 | ASR | 0.4369814886275008 | 0.4475396780811231 |
|  | 0.4390825578437782 | 1998 | ASR | 0.4338757490444319 | 0.4442893666431245 |
|  | 0.4319411940246475 | 1999 | ASR | 0.4268281650010231 | 0.4370542230482719 |
|  | 0.4233332401267823 | 2000 | ASR | 0.4183268599974667 | 0.4283396202560979 |
|  | 0.4061630887965582 | 2001 | ASR | 0.4013135349785925 | 0.411012642614524 |
|  | 0.3901912036909763 | 2002 | ASR | 0.3854916964393094 | 0.3948907109426433 |
|  | 0.3735210828333955 | 2003 | ASR | 0.3689739742953111 | 0.37806819137148 |
|  | 0.3564298753238226 | 2004 | ASR | 0.3520378665309403 | 0.360821884116705 |
|  | 0.3441892422612452 | 2005 | ASR | 0.3399182770434434 | 0.348460207479047 |
|  | 0.3327109053285581 | 2006 | ASR | 0.32855451340056 | 0.3368672972565562 |
|  | 0.3266696607430229 | 2007 | ASR | 0.3225909408441172 | 0.3307483806419287 |
|  | 0.3197093819601497 | 2008 | ASR | 0.3157152144919981 | 0.3237035494283013 |
|  | 0.313776166298861 | 2009 | ASR | 0.3098553778337025 | 0.3176969547640194 |
|  | 0.3109840411579717 | 2010 | ASR | 0.3071159617457653 | 0.314852120570178 |
|  | 0.3055726924992671 | 2011 | ASR | 0.3017724218883815 | 0.3093729631101527 |
|  | 0.2992850610189214 | 2012 | ASR | 0.2955549569297855 | 0.3030151651080573 |
|  | 0.2971452335684771 | 2013 | ASR | 0.293457971336022 | 0.3008324958009322 |
|  | 0.2961499598161573 | 2014 | ASR | 0.2924970824058831 | 0.2998028372264314 |
|  | 0.2946698631921391 | 2015 | ASR | 0.2910514642079757 | 0.2982882621763025 |
|  | 0.2959990807679624 | 2016 | ASR | 0.2923944561583117 | 0.2996037053776132 |
|  | 0.2976169428871323 | 2017 | ASR | 0.2940242758857848 | 0.3012096098884798 |
|  | 0.2974999526318538 | 2018 | ASR | 0.2939298449225766 | 0.301070060341131 |
|  | 0.2984622825183972 | 2019 | ASR | 0.2949061325004373 | 0.3020184325363571 |
|  | 0.2983629315835036 | 2020 | ASR | 0.2948229100294778 | 0.3019029531375295 |
|  | 0.2994660590600078 | 2021 | ASR | 0.2958700022400792 | 0.3030621158799364 |
|  | 0.2923681931516273 | 2022 | ASR | 0.2766751592458464 | 0.3080612270574081 |
|  | 0.2872316022079648 | 2023 | ASR | 0.267392476427348 | 0.3070707279885815 |
|  | 0.2821877567715921 | 2024 | ASR | 0.2590143802233956 | 0.3053611333197887 |
|  | 0.2771091938143947 | 2025 | ASR | 0.2511016584556139 | 0.3031167291731755 |
|  | 0.2720642694967145 | 2026 | ASR | 0.2435361672546364 | 0.3005923717387926 |
|  | 0.2670930595716351 | 2027 | ASR | 0.236232910812933 | 0.2979532083303372 |
|  | 0.2622118930714334 | 2028 | ASR | 0.2292363044828189 | 0.2951874816600479 |
|  | 0.2572936775118038 | 2029 | ASR | 0.2224131611649361 | 0.2921741938586714 |
|  | 0.2522807279566809 | 2030 | ASR | 0.2156717484705502 | 0.2888897074428116 |
|  | 0.2473182821399917 | 2031 | ASR | 0.2090827983891567 | 0.2855537658908266 |
|  | 0.2424735914814683 | 2032 | ASR | 0.2026460254545748 | 0.2823011575083618 |
|  | 0.2377764576350985 | 2033 | ASR | 0.1964154679934122 | 0.2791374472767847 |
|  | 0.2330869077140022 | 2034 | ASR | 0.1902636187560675 | 0.2759101966719369 |
|  | 0.2283051069756073 | 2035 | ASR | 0.1840760427223325 | 0.2725341712288821 |
|  | 0.223519955556558 | 2036 | ASR | 0.1778791423415287 | 0.2691607687715874 |
|  | 0.2187763529800271 | 2037 | ASR | 0.1716578257071051 | 0.2658948802529492 |
|  | 0.2141180856445941 | 2038 | ASR | 0.1654535198302289 | 0.2627826514589594 |
|  | 0.209486360561881 | 2039 | ASR | 0.1591940556279963 | 0.2597786654957657 |
|  | 0.2048438800571194 | 2040 | ASR | 0.1528051278422919 | 0.2568826322719469 |
|  | 0.2002585640674365 | 2041 | ASR | 0.1462851461882803 | 0.2542319819465927 |
|  | 0.195748712276155 | 2042 | ASR | 0.1395967835199828 | 0.2519006410323273 |
|  | 0.1913310734176893 | 2043 | ASR | 0.1327534275533433 | 0.2499087192820353 |
|  | 0.1869567561653239 | 2044 | ASR | 0.1256975279999479 | 0.2482159843306999 |
|  | 0.182593350704876 | 2045 | ASR | 0.1183723825565231 | 0.2468143188532289 |
|  | 0.1782897420279274 | 2046 | ASR | 0.1107824835558556 | 0.2457970004999992 |
|  | 0.174065929743532 | 2047 | ASR | 0.1029208969410746 | 0.2452109625459895 |
|  | 0.1699523983021181 | 2048 | ASR | 0.09483342275501877 | 0.2450713738492175 |
|  | 0.1659228915221225 | 2049 | ASR | 0.08650757084617507 | 0.2453382121980699 |
|  | 0.1619528560900626 | 2050 | ASR | 0.07792399924148152 | 0.2459817129386437 |
| male |  |  |  |  |  |
|  | 0.6400450775623654 | 1990 | ASR | 0.6303134250144413 | 0.6497767301102895 |
|  | 0.6512243596071742 | 1991 | ASR | 0.6417700348746307 | 0.6606786843397177 |
|  | 0.6540242323952484 | 1992 | ASR | 0.6446599834884962 | 0.6633884813020007 |
|  | 0.6522490503968511 | 1993 | ASR | 0.6429933158559666 | 0.6615047849377356 |
|  | 0.646380435398507 | 1994 | ASR | 0.6372673572175523 | 0.6554935135794616 |
|  | 0.6426313881367919 | 1995 | ASR | 0.6336299585795369 | 0.6516328176940469 |
|  | 0.6369777022944783 | 1996 | ASR | 0.6281040965142356 | 0.6458513080747209 |
|  | 0.6320017699117367 | 1997 | ASR | 0.6232457705945814 | 0.640757769228892 |
|  | 0.6276096769244526 | 1998 | ASR | 0.6189707358418259 | 0.6362486180070792 |
|  | 0.6164158082536391 | 1999 | ASR | 0.6079388674513438 | 0.6248927490559343 |
|  | 0.6051100436961838 | 2000 | ASR | 0.5968041002183703 | 0.6134159871739974 |
|  | 0.5820856402777271 | 2001 | ASR | 0.5740294303229163 | 0.590141850232538 |
|  | 0.5620297040473159 | 2002 | ASR | 0.5542032711754115 | 0.5698561369192203 |
|  | 0.5402677951378563 | 2003 | ASR | 0.532678038426917 | 0.5478575518487956 |
|  | 0.5162085128725769 | 2004 | ASR | 0.5088751484160292 | 0.5235418773291245 |
|  | 0.49973454147781 | 2005 | ASR | 0.4925940599272166 | 0.5068750230284034 |
|  | 0.4842774530432952 | 2006 | ASR | 0.4773192184712612 | 0.4912356876153291 |
|  | 0.4772811167976792 | 2007 | ASR | 0.4704382592908193 | 0.484123974304539 |
|  | 0.4683784626591027 | 2008 | ASR | 0.4616680832222609 | 0.4750888420959444 |
|  | 0.4596813513039545 | 2009 | ASR | 0.4530943498825085 | 0.4662683527254006 |
|  | 0.4558615410867048 | 2010 | ASR | 0.4493583401708544 | 0.4623647420025552 |
|  | 0.4486237667862902 | 2011 | ASR | 0.4422258552077653 | 0.4550216783648151 |
|  | 0.4414867179329012 | 2012 | ASR | 0.4351871949658309 | 0.4477862408999714 |
|  | 0.4397448886224 | 2013 | ASR | 0.4335071035235953 | 0.4459826737212046 |
|  | 0.4395144560978118 | 2014 | ASR | 0.4333286213020541 | 0.4457002908935694 |
|  | 0.4385714943897736 | 2015 | ASR | 0.4324339964313906 | 0.4447089923481565 |
|  | 0.4409382249290668 | 2016 | ASR | 0.4348195874454379 | 0.4470568624126956 |
|  | 0.4429503863314685 | 2017 | ASR | 0.4368550126700998 | 0.4490457599928373 |
|  | 0.443249800137591 | 2018 | ASR | 0.4371892810059058 | 0.4493103192692762 |
|  | 0.4449067247328035 | 2019 | ASR | 0.4388677088200998 | 0.4509457406455071 |
|  | 0.4444749071543862 | 2020 | ASR | 0.4384613832000031 | 0.4504884311087693 |
|  | 0.4460407503803247 | 2021 | ASR | 0.4398963686161923 | 0.4521851321444571 |
|  | 0.4357662848593621 | 2022 | ASR | 0.4125143425448071 | 0.459018227173917 |
|  | 0.4285053315880153 | 2023 | ASR | 0.3992418261984132 | 0.4577688369776174 |
|  | 0.4213175233427581 | 2024 | ASR | 0.3871898002765694 | 0.4554452464089468 |
|  | 0.4140918120614719 | 2025 | ASR | 0.3758054555443776 | 0.4523781685785662 |
|  | 0.4068707066847052 | 2026 | ASR | 0.3648737885482209 | 0.4488676248211895 |
|  | 0.3996991912638501 | 2027 | ASR | 0.3542693661629304 | 0.4451290163647698 |
|  | 0.3926082189933367 | 2028 | ASR | 0.3440639473588207 | 0.4411524906278527 |
|  | 0.3854148529828473 | 2029 | ASR | 0.3340674636289032 | 0.4367622423367913 |
|  | 0.3780539904650039 | 2030 | ASR | 0.3241660653856473 | 0.4319419155443605 |
|  | 0.3706694306668756 | 2031 | ASR | 0.3144117268031263 | 0.426927134530625 |
|  | 0.3633434311249615 | 2032 | ASR | 0.3048025424541976 | 0.4218843197957254 |
|  | 0.3561717045578262 | 2033 | ASR | 0.2954668268960365 | 0.4168765822196159 |
|  | 0.3489502227674222 | 2034 | ASR | 0.2862247424062213 | 0.4116757031286231 |
|  | 0.341534324158235 | 2035 | ASR | 0.2769177871095362 | 0.4061508612069338 |
|  | 0.3340129116005112 | 2036 | ASR | 0.2675618868880122 | 0.4004639363130101 |
|  | 0.3264618059052907 | 2037 | ASR | 0.2581572010659031 | 0.3947664107446782 |
|  | 0.3190252191550771 | 2038 | ASR | 0.2488367439410553 | 0.389213694369099 |
|  | 0.311616331691581 | 2039 | ASR | 0.2395036662718973 | 0.3837289971112648 |
|  | 0.3041682890134714 | 2040 | ASR | 0.2300514741256452 | 0.3782851039012977 |
|  | 0.2967446010352291 | 2041 | ASR | 0.2204629954985894 | 0.3730262065718687 |
|  | 0.2893711976276778 | 2042 | ASR | 0.2106945203690432 | 0.3680478748863124 |
|  | 0.2821347404574798 | 2043 | ASR | 0.2008132796248862 | 0.3634562012900735 |
|  | 0.2749613190410421 | 2044 | ASR | 0.1907328891653525 | 0.3591897489167317 |
|  | 0.2677936639747602 | 2045 | ASR | 0.1803636274483567 | 0.3552237005011637 |
|  | 0.2606882375160133 | 2046 | ASR | 0.1697008545917814 | 0.3516756204402452 |
|  | 0.253681254669146 | 2047 | ASR | 0.1587384024105936 | 0.3486241069276985 |
|  | 0.2468605629043714 | 2048 | ASR | 0.1475659866204141 | 0.3461551391883286 |
|  | 0.2401841381140031 | 2049 | ASR | 0.1361558100770693 | 0.3442124661509368 |
|  | 0.2336041507619057 | 2050 | ASR | 0.1244678054630052 | 0.3427404960608061 |
| female |  |  |  |  |  |
|  | 0.2643307858938361 | 1990 | ASR | 0.2583459083495327 | 0.2703156634381394 |
|  | 0.2666123449832931 | 1991 | ASR | 0.2609631923805824 | 0.2722614975860038 |
|  | 0.265641349589504 | 1992 | ASR | 0.2600756820470004 | 0.2712070171320076 |
|  | 0.2649864125777396 | 1993 | ASR | 0.2594786570991391 | 0.27049416805634 |
|  | 0.2622742136874247 | 1994 | ASR | 0.2568621244899481 | 0.2676863028849013 |
|  | 0.2594707457287952 | 1995 | ASR | 0.2541351629547357 | 0.2648063285028547 |
|  | 0.2546359598205933 | 1996 | ASR | 0.2494037327388979 | 0.2598681869022887 |
|  | 0.2501974881985817 | 1997 | ASR | 0.2450612461564472 | 0.2553337302407162 |
|  | 0.2479211740454127 | 1998 | ASR | 0.2428619500815395 | 0.2529803980092858 |
|  | 0.2446643697456452 | 1999 | ASR | 0.2396840103478283 | 0.2496447291434621 |
|  | 0.2386487027826316 | 2000 | ASR | 0.2337835147995619 | 0.2435138907657013 |
|  | 0.2282548315144315 | 2001 | ASR | 0.2235597877918199 | 0.2329498752370431 |
|  | 0.2169509945956936 | 2002 | ASR | 0.2124346976011664 | 0.2214672915902209 |
|  | 0.2058166996654807 | 2003 | ASR | 0.2014793515907824 | 0.2101540477401791 |
|  | 0.1961738783304627 | 2004 | ASR | 0.1919952781660998 | 0.2003524784948257 |
|  | 0.1883499661781864 | 2005 | ASR | 0.1842999616015154 | 0.1923999707548573 |
|  | 0.1813542904120686 | 2006 | ASR | 0.1774177495299958 | 0.1852908312941415 |
|  | 0.1761254953873617 | 2007 | ASR | 0.1722840669172025 | 0.1799669238575209 |
|  | 0.1712189298611974 | 2008 | ASR | 0.1674821324233156 | 0.1749557272990792 |
|  | 0.1680468847755101 | 2009 | ASR | 0.1643763721548382 | 0.171717397396182 |
|  | 0.1660553112333365 | 2010 | ASR | 0.1624368733843992 | 0.1696737490822737 |
|  | 0.162687716998691 | 2011 | ASR | 0.1591388662517957 | 0.1662365677455863 |
|  | 0.1579501856816141 | 2012 | ASR | 0.1544837723431318 | 0.1614165990200964 |
|  | 0.1553097672297446 | 2013 | ASR | 0.1519000784004156 | 0.1587194560590736 |
|  | 0.1533489073095285 | 2014 | ASR | 0.1499892216032989 | 0.1567085930157582 |
|  | 0.1515976085556366 | 2015 | ASR | 0.1482806344150711 | 0.1549145826962021 |
|  | 0.1516487751013146 | 2016 | ASR | 0.1483481455655111 | 0.154949404637118 |
|  | 0.1524366043586338 | 2017 | ASR | 0.1491410526622035 | 0.155732156055064 |
|  | 0.1520993076452939 | 2018 | ASR | 0.1488236990325482 | 0.1553749162580396 |
|  | 0.1523007468910108 | 2019 | ASR | 0.1490335175929643 | 0.1555679761890573 |
|  | 0.1524475451022391 | 2020 | ASR | 0.1491755029019975 | 0.1557195873024807 |
|  | 0.1525269770333859 | 2021 | ASR | 0.1490749260981366 | 0.1559790279686352 |
|  | 0.1491123600584364 | 2022 | ASR | 0.1404912673878441 | 0.1577334527290287 |
|  | 0.1462436554320986 | 2023 | ASR | 0.1352919985616572 | 0.15719531230254 |
|  | 0.1434966890832887 | 2024 | ASR | 0.130690803321528 | 0.1563025748450495 |
|  | 0.140795084456436 | 2025 | ASR | 0.1264163439327131 | 0.1551738249801588 |
|  | 0.1381553645039627 | 2026 | ASR | 0.1223666536155721 | 0.1539440753923533 |
|  | 0.1355476445686349 | 2027 | ASR | 0.118437684182524 | 0.1526576049547458 |
|  | 0.1329535869065591 | 2028 | ASR | 0.1146402081372849 | 0.1512669656758334 |
|  | 0.1303701491735317 | 2029 | ASR | 0.1109551440487494 | 0.1497851542983141 |
|  | 0.127814974710114 | 2030 | ASR | 0.1073590956308026 | 0.1482708537894254 |
|  | 0.1253762698598165 | 2031 | ASR | 0.103876796631606 | 0.1468757430880269 |
|  | 0.1230669736770876 | 2032 | ASR | 0.1004685283415634 | 0.1456654190126118 |
|  | 0.1208427722043198 | 2033 | ASR | 0.09710819554636184 | 0.1445773488622777 |
|  | 0.1186674039984808 | 2034 | ASR | 0.09374624534975878 | 0.1435885626472028 |
|  | 0.1165194838713532 | 2035 | ASR | 0.09032732021466569 | 0.1427116475280407 |
|  | 0.1144368145975707 | 2036 | ASR | 0.08683532418771926 | 0.142038305007422 |
|  | 0.1124133842651869 | 2037 | ASR | 0.08322011078683769 | 0.1416066577435361 |
|  | 0.1104057815095583 | 2038 | ASR | 0.07944295312064739 | 0.1413686098984692 |
|  | 0.1084092544529589 | 2039 | ASR | 0.07547679812464425 | 0.1413417107812735 |
|  | 0.1064390822231571 | 2040 | ASR | 0.07130015846967439 | 0.1415780059766397 |
|  | 0.1045375717036204 | 2041 | ASR | 0.06691219649211619 | 0.1421629469151246 |
|  | 0.1027096143439448 | 2042 | ASR | 0.06229217886247003 | 0.1431270498254196 |
|  | 0.1009134867416367 | 2043 | ASR | 0.05741790731678753 | 0.1444090661664859 |
|  | 0.09913492006166501 | 2044 | ASR | 0.05227766104489202 | 0.145992179078438 |
|  | 0.09737621788225723 | 2045 | ASR | 0.04686673425057074 | 0.1478857015139437 |
|  | 0.09566357358520076 | 2046 | ASR | 0.04120001729702832 | 0.1501271298733732 |
|  | 0.09400269740169016 | 2047 | ASR | 0.03528340484944405 | 0.1527219899539363 |
|  | 0.09237417021742415 | 2048 | ASR | 0.02912559475008178 | 0.1556227456847665 |
|  | 0.09076958759766178 | 2049 | ASR | 0.02273493601207864 | 0.1588042391832449 |
|  | 0.0891929518682768 | 2050 | ASR | 0.01612460413357301 | 0.1622612996029806 |


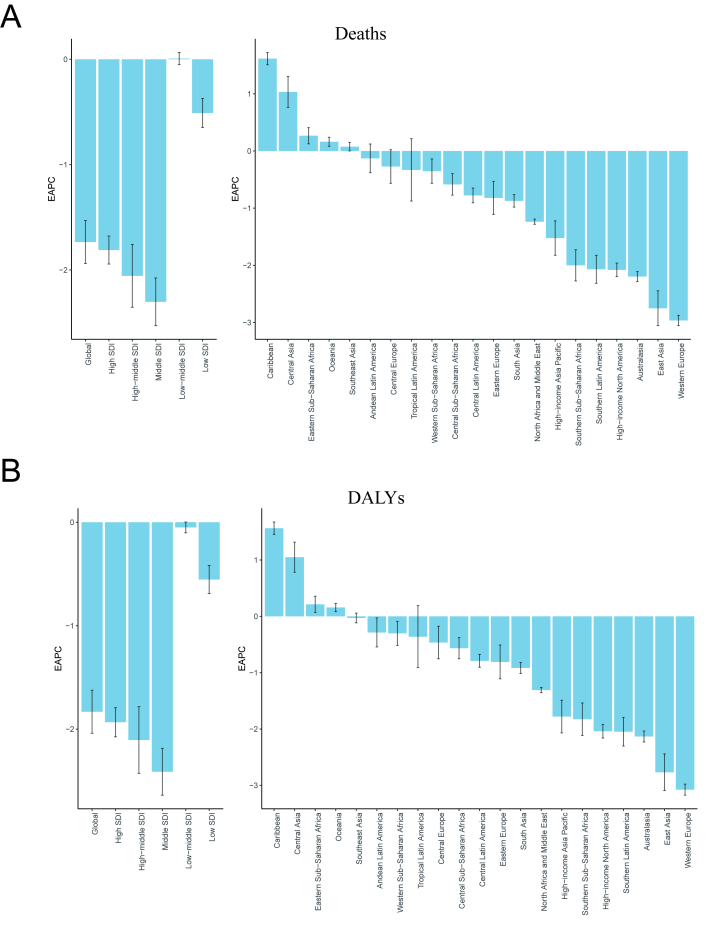


**Figure S1: Trends in EAPC across different SDI regions and 21 GBD regions.** (A) deaths. (B) DALYs.
